# Nectary starch degradation affects nectar chemical composition, but not nectar sugars, in *Arabidopsis thaliana*

**DOI:** 10.1093/plphys/kiaf515

**Published:** 2025-10-15

**Authors:** Erik Martin Solhaug, Michelle Roulier, Hongyuan Zhang, Martina Zanella, Samuel Christian Zeeman, Diana Santelia

**Affiliations:** Department of Environmental Systems Sciences, Institute of Integrative Biology, ETH Zürich, Zürich 8092, Switzerland; Insitute of Molecular Plant Biology, ETH Zürich, Zürich 8093, Switzerland; Department of Environmental Systems Sciences, Institute of Integrative Biology, ETH Zürich, Zürich 8092, Switzerland; Functional Genomics Center Zurich, Zürich 8057, Switzerland; Insitute of Molecular Plant Biology, ETH Zürich, Zürich 8093, Switzerland; Department of Environmental Systems Sciences, Institute of Integrative Biology, ETH Zürich, Zürich 8092, Switzerland

## Abstract

Attracting and rewarding pollinators are important for the reproduction of many flowering plants, and floral nectar plays a central role in plant–pollinator relationships as the primary reward. Nectar production by floral organs called nectaries coincides with a buildup and degradation of nectary starch in many species. While this temporal connection might suggest that nectar sugars are produced from nectary starch, direct evidence to support this idea is lacking. Here, we performed genetic manipulations to test how nectary starch contributes to nectar production in *Arabidopsis* (*Arabidopsis thaliana*). Additionally, we conducted semi-targeted metabolomics experiments to identify which nectar compounds (NCs) depend on nectary starch for their production and secretion. While nectar sugar levels were not consistently lower in plants defective in nectary starch metabolism, mutants producing more nectary starch consistently produced less nectar sugar. We also detected a number of differentially accumulated NCs connected to biotic stress in starch-related mutants, including salicylic acid. Our results suggest that, in *Arabidopsis*, nectary starch is not required as a carbohydrate source to produce nectar sugars per se, but nectary starch metabolism is important for the production and secretion of specialized NCs, which may help nectaries respond to stress. NCs identified from our metabolomics experiment provide the foundation for further investigations into the functional and physiological importance of nectary starch in nectar and floral biology.

## Teaser

Nectary starch influences the production of stress-related nectar compounds in *Arabidopsis*, providing insight into how nectaries metabolize carbon for diverse functions.

## Introduction

Floral nectar is a sugary substance produced by nectaries to reward visiting pollinators. Although major floral attractants include floral display size, color, and scent, these are much more effective if associated with a tangible reward, such as nectar (Bailes et al. 2015; Knauer and Schiestl 2015). Plant–pollinator interactions are of central importance in determining fruit and seed yield in various plant families, including Brassicaceae, Ericaceae, Rosaceae, Solanaceae, and Cucurbitaceae, all of which contain important crop species (Tuell and Isaacs 2010; Motzke et al. 2015; Pereira et al. 2015; Zou et al. 2017; Abrol et al. 2019).

Nectaries of many plant species accumulate starch prior to nectar secretion, and this starch is degraded as nectar sugars are secreted. Starch is a polymer of α-1,4-linked glucose (Glc) units arranged in complex secondary and tertiary structures to form semicrystalline, insoluble granules (Smith and Zeeman 2020). A temporal link between nectar sugar accumulation and nectary starch degradation has been established in diverse species, such as *Nicotiana* spp. (Ren et al. 2007; Liu and Thornburg 2012), squash (*Cucurbita pepo*; Solhaug et al. 2019a,b), and other species in the Rosaceae (Weryszko-Chmielewska et al. 2003; Chwil et al. 2019) and Brassicaceae, including the model species, *Arabidopsis* (*Arabidopsis thaliana*; Davis et al. 1986, 1998; Ren et al. 2007; Lin et al. 2014; Thomas et al. 2017). Adding further support to connect nectary starch degradation with nectar secretion, a number of starch-related genes have been identified in transcriptomic studies in *Arabidopsis* (Kram et al. 2009) and *C. pepo* nectaries (Solhaug et al. 2019b). As soluble sugars can be produced from the direct products of starch degradation, a popular hypothesis has been that nectar sugars are produced from degradation of nectary starch reserves.

*Arabidopsis* flowers possess two types of nectaries, lateral nectaries (LNs), which produce and secrete nectar, and median nectaries, which are nonsecretory (Kram et al. 2009). *Arabidopsis* floral nectar is secreted by three critical metabolic steps, beginning with the synthesis of sucrose (Suc) by Suc phosphate synthase (SPS) and Suc phosphate phosphatase. Indeed, plants silenced in *SUCROSE PHOSPHATE SYNTHASE 1F* (*SPS1F*) and *SUCROSE PHOSPHATE SYNTHASE 2F* (*SPS2F*) are unable to produce floral nectar (Lin et al. 2014). Suc is then transported out of nectar-secreting cells by facilitated diffusion. Mutants defective in Suc uniporter *SUGARS WILL EVENTUALLY BE EXPORTED TRANSPORTER 9* (*SWEET9*) are similarly unable to produce and secrete nectar (Lin et al. 2014). Interestingly, both *sps1f/2f* and *sweet9* mutants showed starch excess phenotypes in the nectaries (Lin et al. 2014), further suggesting a possible connection between nectar sugar production and nectary starch. After Suc is exported by nectar-secreting cells, it is hydrolyzed in the extracellular space by CELL WALL INVERTASE 4 (CWINV4). Although *cwinv4* mutants showed reduced cell-wall invertase activity and were unable to produce nectar, *cwinv4* nectaries did not exhibit a starch excess phenotype in nectaries (Ruhlmann et al. 2010), suggesting that CWINV4 might play an additional role in the degradation of phloem-derived Suc and, thus, in determining the strength of the nectary sink. During nectar secretion, Suc hydrolysis by CWINV4 produces Glc and fructose (Fru), which changes the osmotic potential between the inner cell and the apoplast, leading to an export of water from the nectary parenchyma. These sugars and water then move into the growing nectar droplet through modified nectarostomata (Roy et al. 2017).

The mechanism of nectar sugar production in *Arabidopsis* involving nectary starch breakdown, nectary Suc synthesis, facilitated Suc diffusion, and extracellular Suc hydrolysis is collectively termed the eccrine secretion model. This model is based on bulk flow of sugars from nectary parenchyma cells into the growing nectar droplet (Ren et al. 2007; Lin et al. 2014). Genes orthologous to nectary Suc transporter *SWEET9* are present in eudicots that possess floral nectaries (Lin et al. 2014), suggesting that the eccrine secretion model is conserved among species within this agronomically important clade. Despite the likely common nectar secretion mechanism in eudicots, in pineapple (*Ananas comosus*), a monocot, nectary transcriptional profiling experiments failed to find orthologous genes involved in eccrine-based secretion (*SWEET9* and *CWINV4*; Göttlinger et al. 2024). Similarly, eccrine secretion genes were not identified in nectary transcriptomic studies focused on tulip tree (*Liriodendron tulipifera*), which is a magnoliid (Liu et al. 2019). These two species, and possibly others outside of the eudicot clade, likely utilize alternative secretion mechanisms to produce nectar. The merocrine/granulocrine mechanism is one alternative, involving secretion completed by the fusion of vesicles derived from the endoplasmic reticulum and/or the Golgi apparatus with the plasma membrane, leading to secretion of nectar metabolites. Another alternative, the holocrine secretion mechanism, involves programmed cell death and lysis of nectary parenchyma cells, leading to spillage of the cellular components into the nectar (both reviewed in Roy et al. 2017).

Although some studies have suggested that many species utilize one secretion mechanism, mixed secretion mechanisms have been observed in some cases. For example, in cotton (*Gossypium hirsutum*), transcriptomic studies suggest that *GhSWEET9* is expressed in secretory nectaries; however, ultrastructural experiments revealed abundant endoplasmic reticula in nectaries, supporting a mixed merocrine/eccrine secretion mechanism in this species (Chatt et al. 2021). These models are based on individual plant species or groups of species, so it is possible that some species not yet studied might use a mixture of secretion mechanisms or even a secretion mechanism not yet identified.

The most common sugars present in nectar are Suc, Glc, and Fru (Nicolson 2022), and the proportion of these three sugars can vary according to the species. For example, *C. pepo* flowers produce a nectar rich in Suc (Nepi et al. 2001; Solhaug et al. 2019a), while many flowers in the Brassicaceae family, including *Arabidopsis*, produce a hexose-dominant nectar (Davis et al. 1998), containing nearly all Glc and Fru. This nectar sugar composition in *Arabidopsis* is consistent with a secretion model involving SWEET9 Suc export from nectary parenchyma and CWINV4 hydrolysis of the secreted Suc to Glc and Fru. Although nectars of most species are dominated by sugars, many species also produce nonsugar nectar compounds (NCs), including alkaloids (Kessler et al. 2012; Wright et al. 2013), phenolics (Afik et al. 2006), and various amino acids (Carter et al. 2006; Solhaug et al. 2021), among others (Roy et al. 2017), which can profoundly influence pollinator behavior and visitation. Additionally, defense proteins have been detected in floral nectar, such as lipid-transfer protein 2.1 in field mustard (*Brassica rapa*; BrLTP2.1), which has shown antimicrobial activity and putatively disrupts microbial membranes (Schmitt et al. 2018), or NADPH oxidase in *Nicotiana* LxS8 hybrids, which produces H_2_O_2_ to reduce microbial growth and protect the nectary from infection (Carter et al. 2007). Recent transcriptomic and biochemical studies from eudicots *Arabidopsis* (Kram et al. 2009), dandelion (*Taraxacum spp.*; Claude et al. 2025), desert cherry (*Nitraria tangutorum*; Chen et al. 2021), and *C. pepo* (Solhaug et al. 2019a,b, 2021) have shown that the biosynthesis of NCs occurs within nectaries as part of eccrine nectar secretion, emphasizing the importance of nectary metabolism in actively determining nectar chemical composition.

Despite numerous studies suggesting that nectary starch degradation provides carbon backbones for the production and secretion of nectar sugars, one recent study has called this hypothesis into question. Specifically, in *C. pepo*, the total amount of nectar sugar (micromoles of Glc equivalents in Suc and Glc) was found to be nearly two-fold higher than the micromoles of Glc equivalents accumulated in nectary starch (Solhaug et al. 2019a). Additionally, detaching presecretory *C. pepo* flowers from plants and allowing them to open in water led to lower total nectar sugar compared to undetached flowers, suggesting that phloem-derived sugars contribute substantially to the total nectar sugar produced (Solhaug et al. 2019a).

In this study, we sought to address how nectary starch influences the production and secretion of floral nectar in *Arabidopsis*. *Arabidopsis* plants largely self-pollinate; however, their flowers retain functional nectaries. Although their small nectaries preclude many experiments related to biochemistry and metabolism, various genetic tools, accessible mutants, and ease of transformation make *Arabidopsis* a good model system to address our current research questions with molecular methods. We examined patterns of nectary starch and nectar sugar production in wild-type (WT) plants over the diel cycle and at different flower developmental stages. We also examined nectary starch and nectar sugar production in mutants defective in starch metabolism and created transgenic lines with nectary-specific deregulation of starch metabolism. Finally, we measured NCs with a semi-targeted metabolomics experiment using starch-related mutants and WT plants. Our data suggest that nectary starch alone has limited, if any, influence on the production and secretion of nectar sugars in *Arabidopsis*. Instead, the degradation of nectary starch during secretion likely provides carbon for the synthesis of nonsugar NCs. The NCs identified from our data will inform future studies surrounding the ecophysiological importance of starch in nectaries.

## Results

### Starch and nectar dynamics in WT flowers

**Figure 1.**
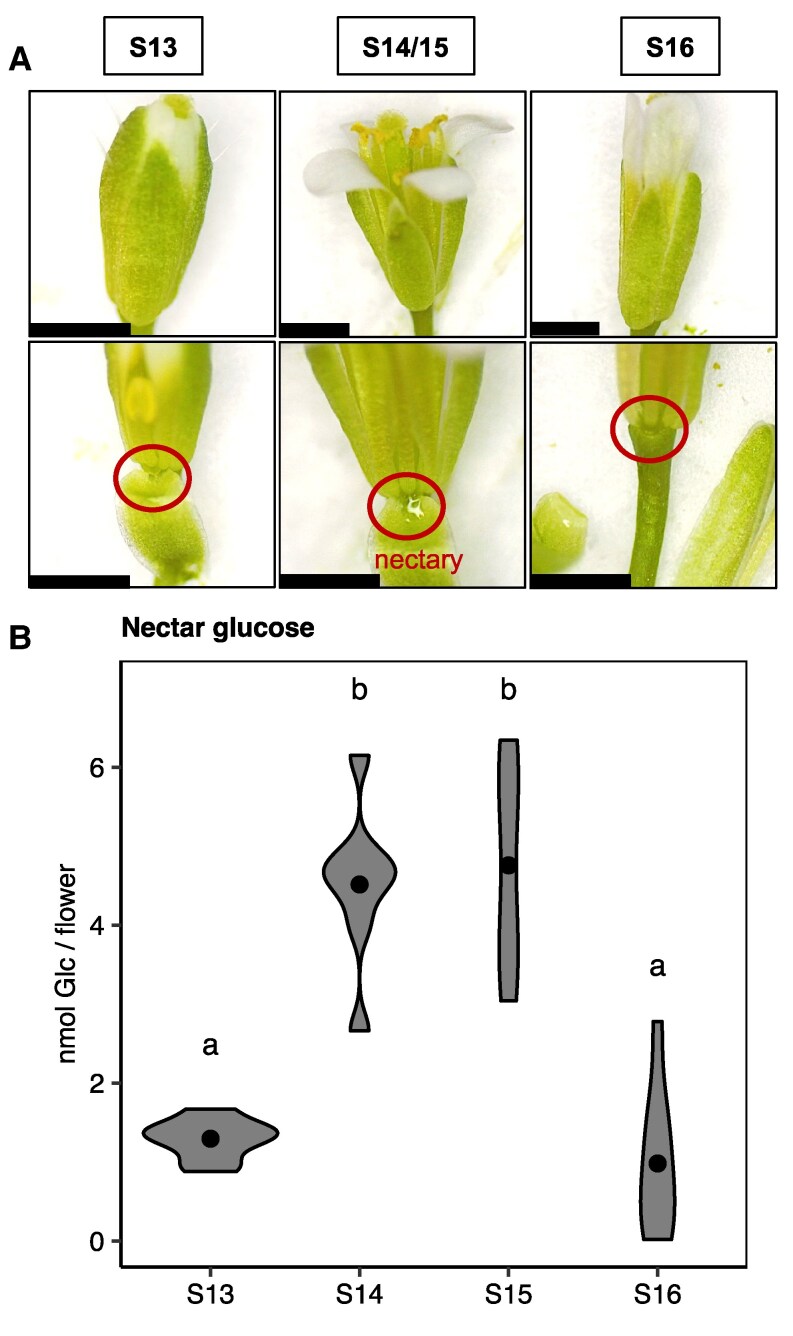
Production of *Arabidopsis* nectar coincides with flower opening. **A)** WT flowers at different developmental stages showing accumulation of nectar (stage 13 = S13, stage 14/15 = S14/S15, stage 16 = S16; scale bar = 1 mm). Two images of the same flower at each stage are depicted, with upper images showing the whole flower, and lower images showing exposed nectaries (sepals peeled back). **B)** Total nectar Glc (nanomoles of Glc per flower) accumulation throughout the development of WT flowers (S13 to S16; between ZT, ZT3, and ZT5). Each biological replicate represents a pooled sample of nectar collected from seven flowers from seven different plants (one flower/plant). Violins represent the distribution of the data, while points represent the average within each group (*N* = 8). These data were from one experiment. Points that share a letter are not significantly different from one another (1-way ANOVA with Tukey's post hoc test, *P* < 0.05). All plants were grown under long-day conditions (16 h light/8 h dark).

In order to better understand the production of floral nectar in *Arabidopsis*, we first examined the diel and developmental fluctuations in nectar sugar. Four different developmental stages were chosen, as described previously (Smyth et al. 1990; Borghi et al. 2022). Briefly, stage 12 (S12) flowers are closed, without petals visible. Stage 13 (S13) flowers are slightly open, with petals visible. Anthesis occurs at S13. Both stage 14 (S14) and stage 15 (S15) flowers are fully open (Smyth et al. 1990; Borghi et al. 2022). In S14 flowers, the long stamens extend above the stigma, while in S15 flowers, the stigma is extended above the stamens. In stage 16 (S16) flowers, the petals and sepals begin to dehisce as the silique develops (Fig. 1A). Nectar was collected at S13 to S16 at the same time of day, at zeitgeber time (ZT) between ZT3 and ZT5 (i.e. 3 and 5 h after dawn), and total nanomoles of nectar Glc per flower were measured. Although the flowers were not fully open, there was 1.3 nmol Glc per flower secreted at S13 (Fig. 1B). From S13 to S14, the amount of nectar Glc increased by more than 3-fold to 4.5 nmol Glc/flower (Fig. 1B) and remained high in S15 flowers. The total nectar Glc then decreased to 1 nmol Glc/flower in S16 flowers (Fig. 1B). Since there was no significant change between the nectar of S14 and S15 flowers, we did not discriminate between flowers of these two stages when collecting nectar from the secretion stage (S14/S15) throughout the rest of this study.

**Figure 2.**
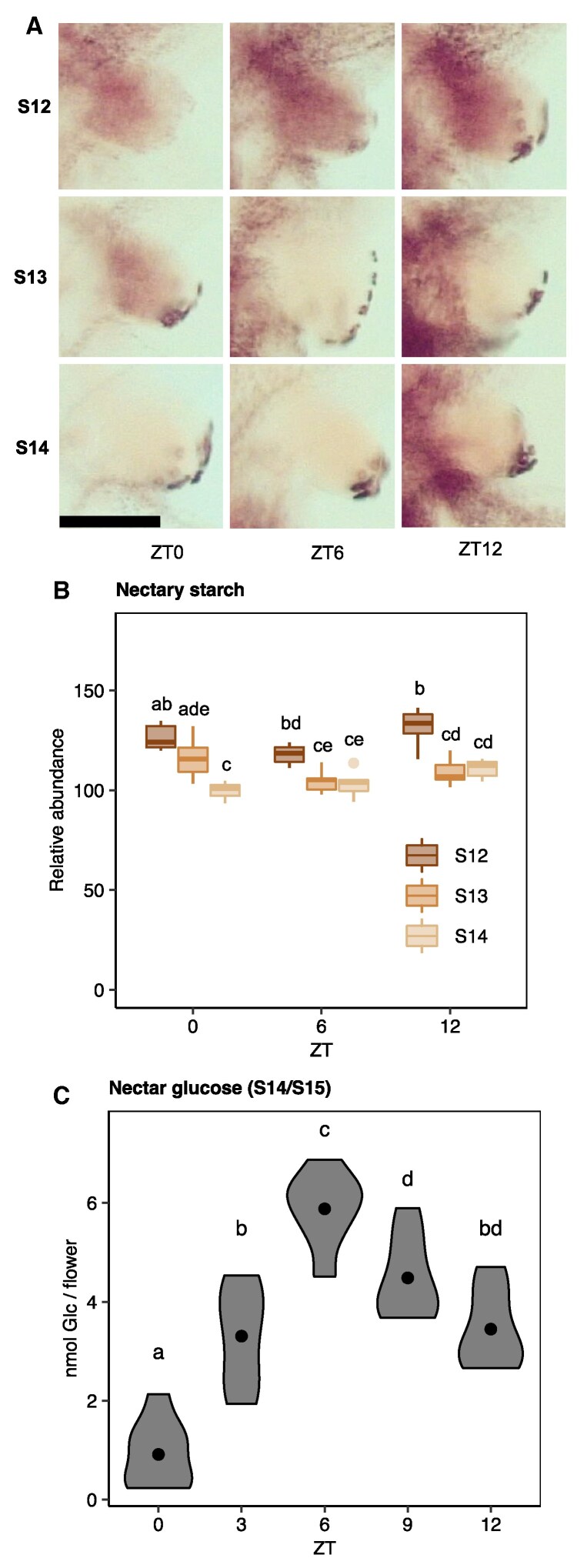
Diel patterns of nectar secretion do not coincide with changes in nectary starch. **A** and **B)** Nectary starch stain images (A) and (B) quantification measured at indicated stages throughout a diel cycle (stage 12 = S12, stage 13 = S13, stage 14 = S14, ZT = zeitgeber time, ZT0 = dawn, ZT6 = 6 h post dawn, ZT12 = 12 h post dawn). Nectary starch was stained with an iodine-based protocol. Each box represents data from six flowers, taken from six different plants (one flower/plant, *N* = 6; two-way ANOVA with Tukey's post hoc test, *P* < 0.05). The center line within each box is the median (Q2), while the box represents the interquartile range (IQR), extending from the first quartile (Q1) to the third quartile (Q3), and the whiskers represent 1.5× the IQR from Q1 and Q3. Outliers are shown as individual points. Scale bar = 100 µm, which applies to all nine nectary images in panel (A). **C)** Total nectar Glc (nanomoles of Glc per flower) accumulation in stage 14/15 (S14/S15) flowers collected throughout a diel cycle (ZT0–ZT12). Each biological replicate represents a pooled sample of nectar collected from seven flowers from seven different plants (one flower/plant). Violins represent the distribution of the data, while points represent the average within each group. Points that share a letter are not significantly different from one another (*N* = 8; 1-way ANOVA with Tukey's post hoc test, *P* < 0.05). These data were from one experiment.

To establish diurnal patterns of starch metabolism in relation to nectar secretion, nectary starch dynamics were first examined in flowers at three timepoints during a diel cycle, ZT0, ZT6, and ZT12. At each timepoint, three developmental stages were examined, including S12, S13, and S14. Each stage encompasses at least 24 h of developmental time (Borghi et al. 2022). Flowers at each stage and timepoint were stained for starch, and the starch content was quantified by staining intensity (Supplementary Fig. S1). For all stages measured, there was a slight increase in starch in the receptacle and pedicel from ZT0 to ZT12 (Supplementary Fig. S2). In a S12 flower, nectary starch did not significantly change from ZT0 to ZT12 (Fig. 2, A and B). In a S13 flower, nectary starch was present at the beginning of the morning (ZT0) as in S12 flowers (Fig. 2B). However, at ZT6 and ZT12, there was significantly less nectary starch in S13 compared to S12 flowers (Fig. 2, A and B). Nectary starch was lower in S14 compared to S12 flowers at all timepoints (Fig. 2, A and B). Together, these data suggest that a large portion of nectary starch degradation occurs between ZT0 and ZT6 in S13 flowers.

To further understand the daily nectar secretion dynamics in *Arabidopsis*, nectar Glc was analyzed throughout a diel cycle in secretory (S14/S15) flowers. Accumulation of nectar followed a distinct diurnal pattern (Fig. 2C). At ZT0, there was 0.9 nmol Glc/flower, which increased steadily to almost 5.9 nmol Glc/flower by ZT6. The amount of nectar then decreased gradually after ZT6 reaching 3.5 nmol Glc/flower by ZT12 (Fig. 2C). Together, these data suggest that nectar sugars are actively produced and secreted during the day in open S14/S15 flowers without marked changes in nectary starch.

Next, transcript levels for three genes (*SPS2F*, *SWEET9*, and *CWINV4*) that have been previously shown to play a critical role in *Arabidopsis* nectar production were examined throughout the day in S14/S15 flowers (Kram et al. 2009; Ruhlmann et al. 2010; Lin et al. 2014). In S14/S15 flowers, the expression of *SPS2F* did not vary throughout the day (Supplementary Fig. S3). However, the expression of *SWEET9* was over two-fold higher at ZT0 compared to ZT6 and ZT12, while the expression of *CWINV4* was nearly five-fold higher at ZT0 compared to ZT6 and ZT12 (Supplementary Fig. S3). Notably, expression of genes known to be required for nectar production peak immediately before the part of the day when nectar accumulation is most rapid.

### Mutations in starch metabolic enzymes do not consistently influence nectar secretion

**Figure 3.**
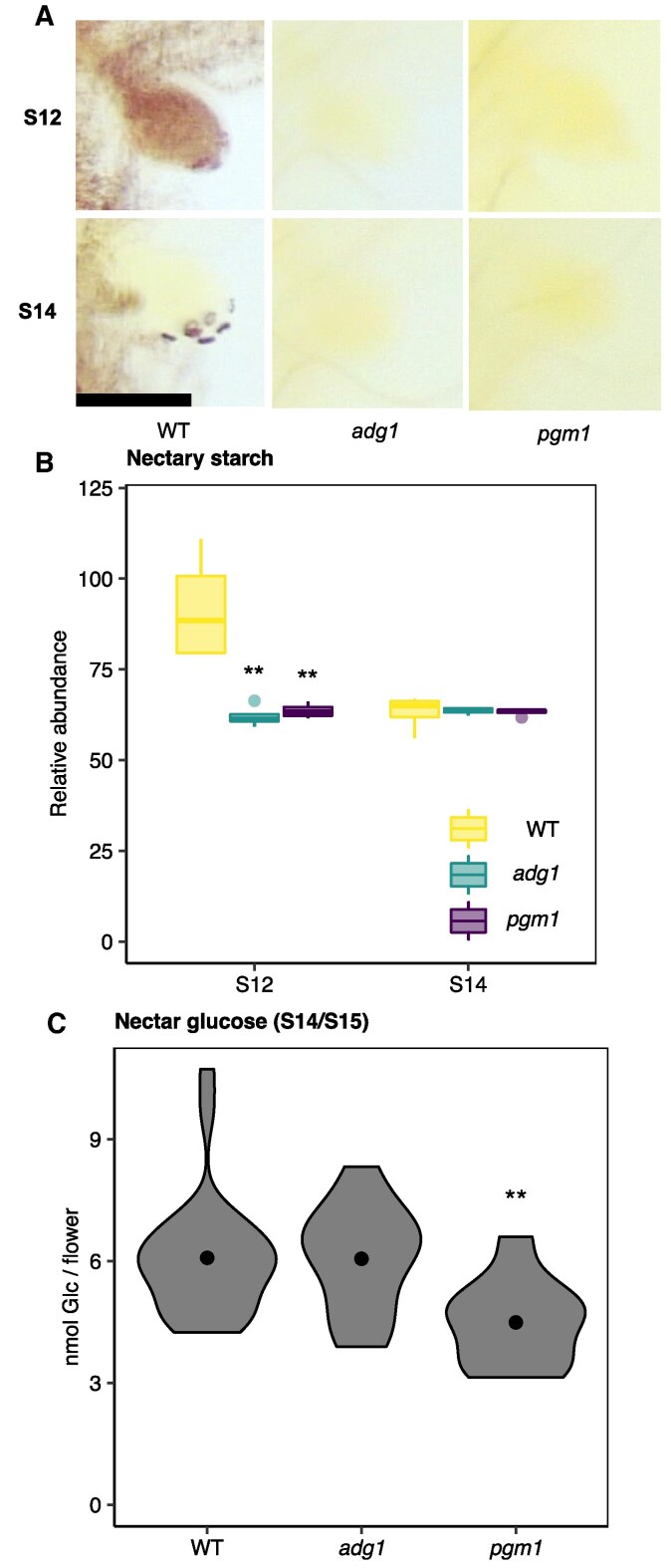
Inability to synthesize starch does not influence nectar sugars. **A** and **B)** Nectary starch stain images (A) and (B) quantification measured at indicated stages (S12–S14) in starch synthesis mutants (*adg1* and *pgm1*) compared to WT with an iodine-based staining protocol. Flowers were collected at ZT6 (ZT6 = zeitgeber time 6, 6 h post dawn). Each box represents data from 4 flowers, taken from 4 different plants (one flower/plant; *N* = 4; two-sample *t*-test, *P* < 0.05). The center line within each box is the median (Q2), while the box represents the interquartile range (IQR), extending from the first quartile (Q1) to the third quartile (Q3), and the whiskers represent 1.5× the IQR from Q1 and Q3. Outliers are shown as individual points. Scale bar = 100 µm, which applies to all six nectary images in panel (A). Data in A and B are from one experiment. **C)** Total nectar Glc (nanomoles of Glc per flower) accumulation in stage 14/15 (S14/S15) flowers collected from starch synthesis mutants. Nectar was collected between the hours of ZT3 and ZT5. Each biological replicate represents a pooled sample of nectar collected from seven flowers from seven different plants (one flower/plant). Violins represent the distribution of the data, while points represent the average within each group. *N* = 26 for WT (3 experiments); *N* = 16 for *adg1* (two experiments); *N* = 10 for *pgm1* (one experiment); two-sample *t*-test, ** = *P* < 0.05. Although data in C are pooled from multiple experiments, statistics were done only between genotypes that were grown together (with the same sample sizes).

To test whether starch is required for the production of nectar sugars, nectar Glc was examined in mutants defective in two starch synthesis genes, *ADP-GLUCOSE PYROPHOSPHORYLASE SMALL SUBUNIT 1* (*ADG1*) and the plastidial *PHOSPHOGLUCOMUTASE1* (*PGM1*). ADP-Glc pyrophosphorylase catalyzes the synthesis of ADP-Glc from Glc-1P, while phosphoglucomutase catalyzes the reversible conversion of Glc-6P to Glc-1P (Streb and Zeeman 2012). Flowers of *adg1* and *pgm1* mutants showed significantly decreased starch in S12 nectaries compared to the WT (Fig. 3, A and B) and visibly reduced starch in whole flowers (Supplementary Fig. S4) at both S12 and S14, which is consistent with the well-established roles that both ADG1 and PGM1 play in starch synthesis (Caspar et al. 1985; Lin et al. 1988). Despite the reduced nectary and floral starch in both *adg1* and *pgm1* mutants, changes in nectar sugar production varied depending on the mutant. Specifically, *pgm1* mutants had a 33% reduction in total nectar Glc compared to WT, while there was no significant difference in nectar Glc between *adg1* mutants and WT (Fig. 3C). Together, these data suggest that floral nectar production in *Arabidopsis* is not consistently affected by defects in whole-plant starch metabolism.

### Nectary-specific silencing of starch synthesis does not consistently impact nectar sugar production

**Figure 4.**
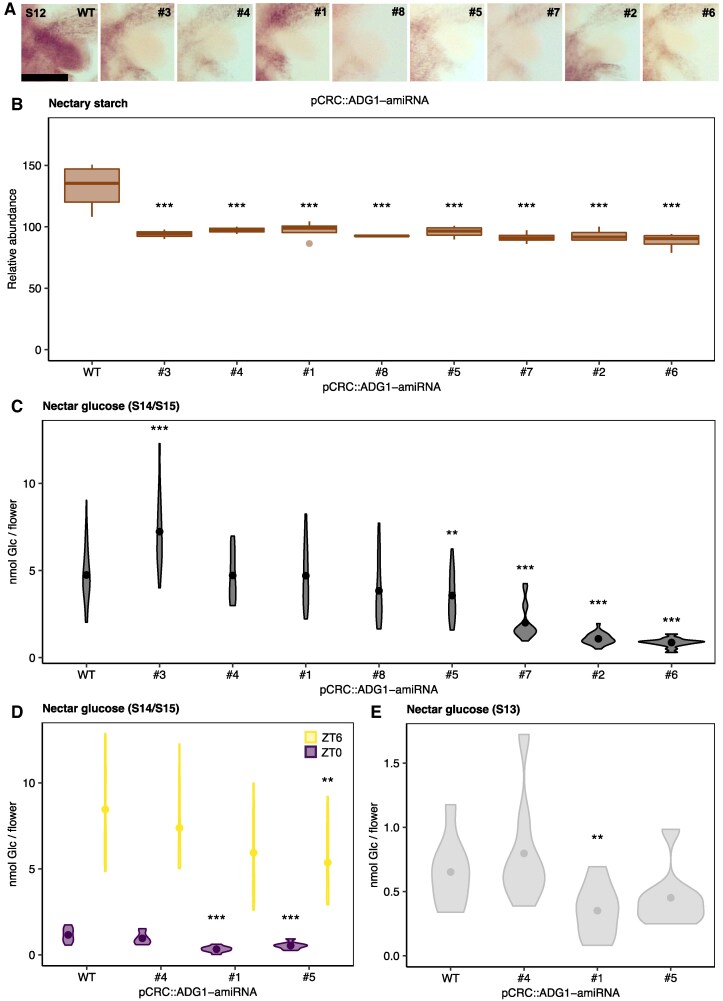
Nectary-specific silencing of *ADG1* leads to reduced nectary starch but inconsistent changes in nectar sugar. **A** and **B)** Nectary starch stain images **(A)** and quantification **(B)** measured at stage 12 (S12) in mutants and WT with an iodine-based staining protocol. Scale bar = 100 µm, which applies to all nine nectary images in panel A. Flowers were collected at ZT6 (ZT = zeitgeber time, ZT6 = 6 h post dawn). Each box represents data from multiple flowers, each taken from different plants (*N* = 11 for WT; *N* = 4 for line #1, line #2, and line #5–line #8; *N* = 3 for line #3 and line #4; two-sample *t*-test, mutant vs. WT, *** = *P* < 0.005). The center line within each box is the median (Q2), while the box represents the interquartile range (IQR), extending from the first quartile (Q1) to the third quartile (Q3), and the whiskers represent 1.5× the IQR from Q1 and Q3. Outliers are shown as individual points. Data in A and B are from one experiment conducted on each line. **C–E)** Total nectar Glc (nanomoles of Glc per flower) accumulation in stage 14/15 flowers collected **(C)** between the hours of ZT3 and ZT5 or **(D)** at different points throughout the day, specifically between the hours of ZT0 and ZT2 (ZT0) and between the hours of ZT6 and ZT8 (ZT6). **E)** Total nectar Glc (nanomoles of Glc per flower) in S13 flowers, collected between the hours of ZT3 and ZT5. Each biological replicate represents a pooled sample of nectar collected from seven flowers from seven different plants (one flower/plant). Violins represent the distribution of the data, while points represent the average within each group. C: *N* = 58 for WT (seven experiments), *N* = 34 for line #1 (four experiments), *N* = 22 for line #2 and line #5 (three experiments), *N* = 16 for line #7 and line #8 (two experiments), and *N* = 14 for line #3, line #4, and line #6 (two experiments); D: *N* = 8 (one experiment) ; E: *N* = 8 (one experiment); two-sample *t*-test, mutant vs. WT, * = *P* < 0.1, ** = *P* < 0.05, *** = *P* < 0.005. Although data in B and C are pooled from multiple experiments, statistics were done only between genotypes that were grown together (with the same sample sizes).

We next wondered whether reduced accumulation of starch in the nectary specifically might impact nectar sugar production. Mutants defective in starch metabolism have shown alterations in levels and export of soluble sugars from the leaves (Kölling et al. 2015), which can influence the supply of phloem-derived sugars to sink tissues such as seeds. For example, reduced seed oil in *pgm1* mutants was shown to be connected to reduced Suc supply during seed-filling (Andriotis et al. 2012). As nectaries are an additional sink, nectar production could be altered by such phloem sugar variation in ways that are difficult to predict. To overcome this limitation, mutants were generated that silenced *ADG1* in the nectaries using an artificial microRNA (amiRNA) expressed under the control of a promoter for *CRABS CLAW* (*CRC*), a gene that is highly expressed and enriched in nectaries (Bowman and Smyth 1999; Kram et al. 2009). A total of eight pCRC::ADG1-amiRNA lines (#1–#8) were analyzed for nectary starch and nectar production (Fig. 4). All eight lines exhibited significant reductions in nectary starch compared to the WT background at S12, with nectaries visibly devoid of starch (Fig. 4, A and B). There was no apparent reduction in starch in other floral tissues such as pedicels, stamens, or ovules (Supplementary Fig. S5) nor in leaves (Supplementary Fig. S6A), suggesting *ADG1* silencing was specific to the nectaries. *ADG1-amiRNA* transcript levels were similar, while *ADG1* transcripts were consistently two-fold lower in whole flowers of all lines examined compared to the WT, suggesting comparable levels of *ADG1* silencing (Supplementary Fig. S6B). *ADG1* expression was also examined in the leaves of WT and pCRC::ADG1-amiRNA mutant lines (line #1 through line #4), but as expected, there was no change in the expression of *ADG1* (Supplementary Fig. S6C). Despite the similar absence of nectary starch in all lines analyzed, changes in nectar Glc were surprisingly inconsistent. Four of the eight lines (#2, #5, #6, and #7; Fig. 4C) had a significant reduction in nectar Glc, with lines #2 and #6 being most severely affected (4.3-fold and 5.4-fold lower than the WT, respectively). However, three of the eight lines (#1, #4, and #8) had no significant change in nectar sugar, and line #3 actually had 1.5-fold higher nectar sugar than the WT (Fig. 4C).

To test whether pCRC::ADG1-amiRNA mutants might have alterations in either early (S13) nectar secretion or diurnal fluctuations in nectar Glc levels, we examined the nectar Glc in the WT, line #1, line #4, and line #5 in ZT0 and ZT6 flowers (S14/S15). Line #1 and line #5 showed over two-fold reduced nectar Glc at ZT0 compared to the WT (Fig. 4D); however, line #4 had the same nectar Glc as WT at ZT0 (Fig. 4D). At ZT6, the nectar Glc was not significantly different for line #4 and line #1, while line #5 had a significant reduction in nectar Glc compared to the WT (Fig. 4D). We next examined the production of nectar in immature (S13) flowers, collected at mid-morning (ZT3–ZT5). Although line #1 had significantly less nectar, there was no change in S13 nectar Glc for line #4 or line #5 compared to the WT (Fig. 4E). To further probe the mechanism behind inconsistent differences in nectar Glc, we next examined the expression of nectary carbon metabolism genes, including *SPS2F*, *SWEET9*, and *CWINV4* in pCRC::ADG1-amiRNA lines and WT flowers. All of the pCRC::ADG1-amiRNA lines examined had significantly lower *CWINV4* expression, and all but one (line #6) had significantly lower *SPS2F* expression compared to the WT (Supplementary Fig. S7). For *SWEET9* expression, lines #2 and #6 had >5-fold reductions, line #5 had a >2-fold reduction, whereas lines #4 and #1 had a mild (∼28%) or no reduction compared to the WT. Strikingly, the patterns of *SWEET9* expression across different pCRC::ADG1-amiRNA lines were closely aligned with the severity of nectar Glc reductions, suggesting that nectar Glc variability in the independent pCRC::ADG1-amiRNA lines might be explained by differential *SWEET9* expression.

### Mis-regulation of AGPase leads to a consistent reduction in nectar sugar

Defects in starch synthesis, presumably leading to reduced carbon derived from nectary starch, did not lead to consistent changes in nectar Glc. However, if the production of nectar Glc is influenced (but not limited) by nectary starch, increased accumulation of nectary starch might lead to more starch degradation products, possibly resulting in more nectar sugar. To test this hypothesis, we used a modified AGPase small subunit from *Escherichia coli*, called *Glycogen C Triple Mutant* (*GlgC-TM*). GlgC-TM contains mutations in regulatory domains that render the enzyme insensitive to allosteric regulation and constitutively active. Indeed, expression of *GlgC-TM* in the leaves of *Arabidopsis* was previously shown to increase starch content by stimulating starch synthesis (Mugford et al. 2014). We thus generated transgenic lines expressing the *GlgC-TM* sequence fused to the chloroplastic-targeting sequence and 3′ UTR from *Arabidopsis ADG1* under the control of the *CRC* promoter (pCRC::GlgC-TM).

**Figure 5.**
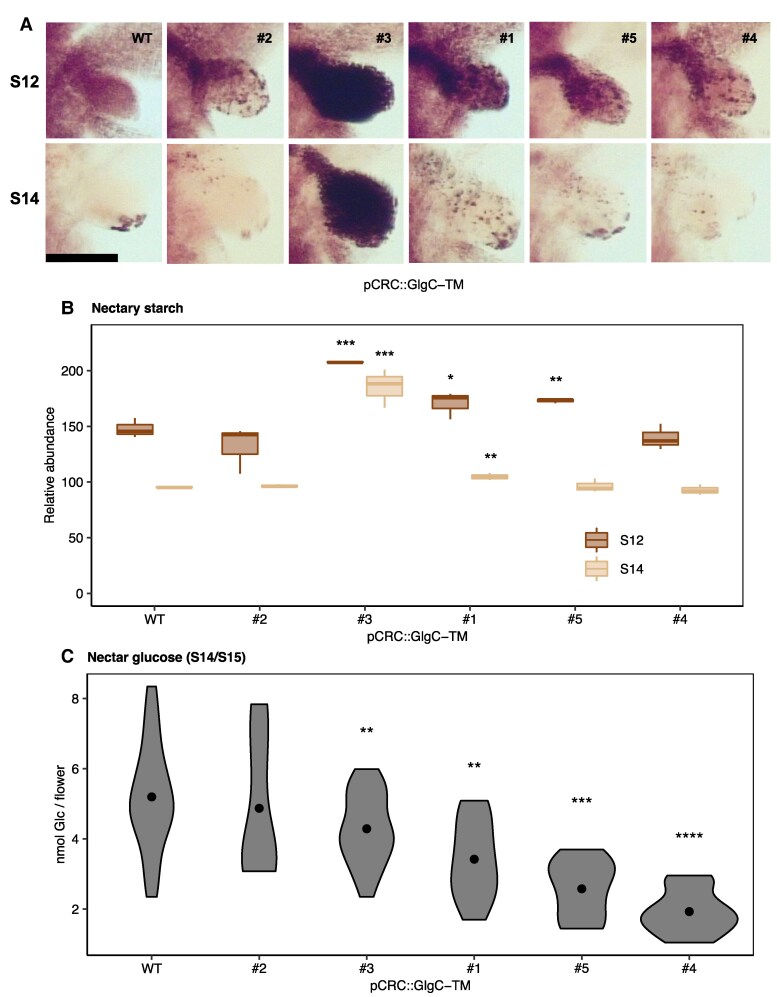
Nectary-specific expression of modified *E. coli* starch synthesis gene *GlgC-TM* leads to inconsistent changes to nectary starch, but lower nectar sugar. **A** and **B)** Nectary starch stain images **(A)** and nectary stain quantification **(B)** measured at indicated stages (stage 12 = S12, stage 14 = S14) in mutants and WT with an iodine-based staining protocol. Flowers were collected at ZT6 (ZT = zeitgeber time, ZT6 = 6 h post dawn). (*N* = 3; two-sample *t*-test, mutant vs. WT, * = *P* < 0.1, ** = *P* < 0.05, *** = *P* < 0.005). The center line within each box is the median (Q2), while the box represents the interquartile range (IQR), extending from the first quartile (Q1) to the third quartile (Q3), and the whiskers represent 1.5× the IQR from Q1 and Q3. Outliers are shown as individual points. Scale bar = 100 µm, which applies to all twelve nectary images in panel A. Data in A and B are from one experiment conducted on each line. **C)** Total nectar Glc (nanomoles of Glc per flower) in stage 14/15 (S14/S15) flowers collected between the hours of ZT3 and ZT5. Each biological replicate represents a pooled sample of nectar collected from seven flowers from seven different plants (one flower/plant). Violins represent the distribution of the data, while points represent the average within each group. **C)**  *N* = 20 for WT and line #3 (three experiments), *N* = 12 for line #1, #2, #4, and line #5 (two experiments); two-sample *t*-test, mutant vs. WT, * = *P* < 0.1, ** = *P* < 0.05, *** = *P* < 0.005. Although data in C are pooled from multiple experiments, statistics were done only between genotypes that were grown together (with the same sample sizes).

Three of the pCRC::GlgC-TM lines (#3, #1, and #5) had significantly higher nectary starch compared to WT at S12, while lines #2 and #4 accumulated WT levels of nectary starch (Fig. 5, A and B). Qualitatively, the nectary starch in all pCRC::GlgC-TM lines formed spot-like structures, possibly representing amyloplasts or large starch granules (Fig. 5A and Supplementary Fig. S8), in contrast to the relatively uniform staining of WT nectaries. At S14, pCRC::GlgC-TM lines #3 and #1 both had significantly higher nectary starch, while the other lines (#2, #4, and #5) had no significant change compared to the WT (Fig. 5, A and B).

Surprisingly, nectar Glc was significantly lower in 4 out of 5 lines measured (lines #1, #3, #4, and #5; Fig. 5C) while for line #2, there was no significant difference. To understand whether differences in nectary starch between the different lines were associated with the levels of *GlgC-TM* or *ADG1* transcripts, we examined gene expression with RT-qPCR. There was no significant difference in expression of *GlgC-TM* between lines #2, #3, #1, and #5 (Supplementary Fig. S9A). However, there was a consistent reduction in expression of *ADG1* in flowers of all independent lines (Supplementary Fig. S9A).

To determine whether the pCRC::GlgC-TM construct was expressed outside of the nectary, we measured its expression in leaves. Although we detected the expression of *GlgC-TM* in line #1, the expression level was not significantly different to the background signal obtained for the WT (Supplementary Fig. S9B), suggesting that the *GlgC-TM* transcript is not present in the pCRC::GlgC-TM leaves. Additionally, there was no increase in starch in the leaves of line #1; rather, there was an over 1.5-fold decrease in leaf starch for all five lines measured (Supplementary Fig. S10). Within flowers, increased starch accumulation in pCRC::GlgC-TM lines was limited to nectaries (see line #3, Supplementary Fig. S8D), further demonstrating the specificity of the pCRC::GlgC-TM construct. Together, the data from lines expressing nectary-enriched *GlgC-TM* suggest that introducing a misregulated AGPase into nectaries has an inconsistent effect on nectary starch accumulation but leads to a consistent reduction in nectar Glc.

### Nectar metabolomics reveals possible starch-derived metabolites

Since our evidence suggested that nectary starch degradation does not contribute to the production of nectar Glc, we hypothesized that starch may play a role in the production and secretion of specific nonsugar NCs. To test this hypothesis, we determined whether mutants defective in nectary starch accumulation had alterations in global nectar metabolites. Three pCRC::ADG1-amiRNA lines (lines #1, #2, and #5) were specifically chosen to represent variability in nectar Glc levels. Consistent NC changes in these lines would suggest that reduced nectary starch led to altered NC accumulation in spite of any potential differences in nectar volume, which we could not directly measure due to the small size of *Arabidopsis* flowers.

**Figure 6.**
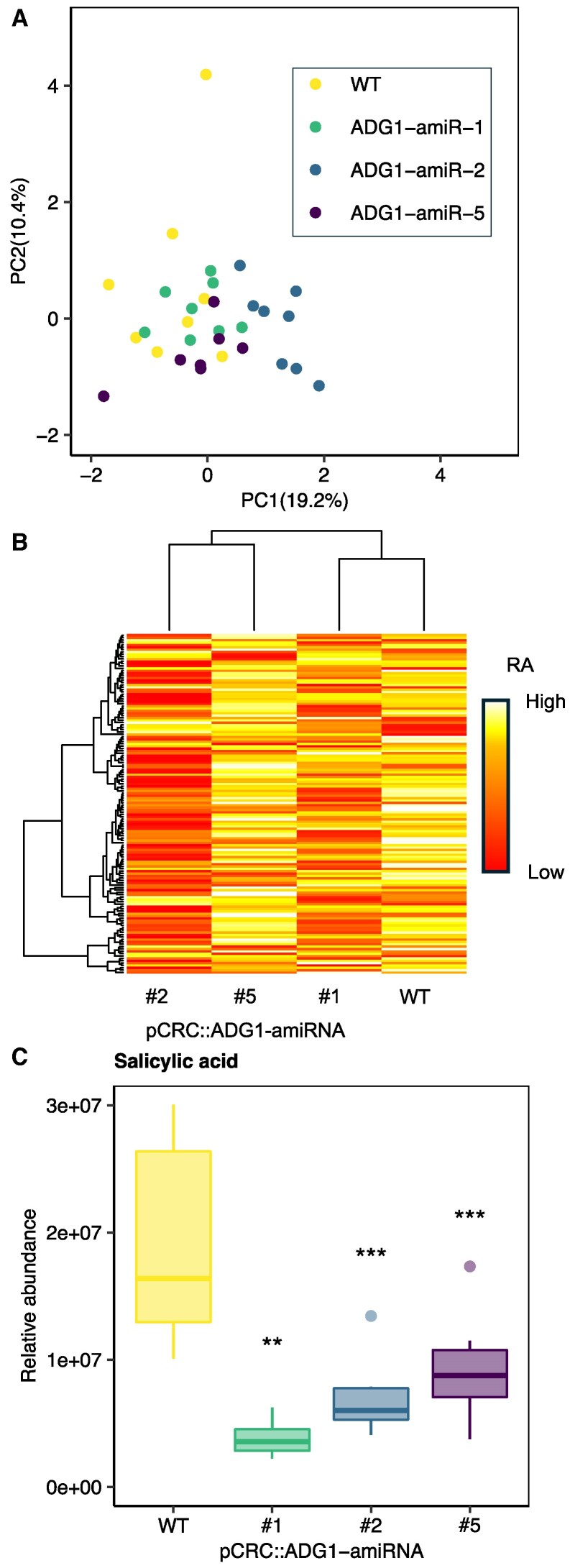
Metabolite profile of nectar suggests some differences in NCs between WT and pCRC::ADG1-amiRNA mutants. **A)** PCA and **B)** heatmap of mean relative abundances (RAs) depicting hierarchical clustering using data from all 150 metabolites measured (white = highest, yellow = mid-high, orange = mid-low, red = lowest). **C)** RA of SA in nectar of mutants and WT (*N* = 8; two-sample *t*-test, mutant vs. WT, * = *P* < 0.1, ** = *P* < 0.05, *** = *P* < 0.005). The center line within each box is the median (Q2), while the box represents the interquartile range (IQR), extending from the first quartile (Q1) to the third quartile (Q3), and the whiskers represent 1.5 × the IQR from Q1 and Q3. Outliers are shown as individual points. Each biological replicate represents a pooled sample of nectar collected from seven flowers from seven different plants (one flower/plant). Metabolites were measured using LC/MS-MS as described in methods. Plants were grown under long-day (LD) conditions (16 h light/8 h dark), and nectar was harvested from stage 14/15 (S14/S15) flowers at ZT3–ZT5 (ZT = zeitgeber time, ZT3 = 3 h post dawn).

We used Glc as a reference compound and observed the same general pattern in the metabolomic data (Table 1) compared to our enzymatic measurements of these samples (Supplementary Fig. S11). Specifically, for this subset of data (which were also included in the pooled nectar data for all pCRC::ADG1-amiRNA lines of Fig. 4C), there was no change in nectar Glc in lines #1 and #5, whereas line #2 had a drastic decrease in total nectar Glc compared to the WT (Supplementary Fig. S11). NCs were measured in pCRC::ADG1-amiRNA lines and the WT using LC-MS/MS, with a method that preferentially detected polar metabolites. In total, 150 unique metabolites were detected using two method modes, positive and negative. The top compounds significantly different from WT in the transgenic lines were included, with the most likely identification of each compound based on retention time (RT) and mass-to-charge ratio (*m*/*z*; Table 1). All measured metabolites were used in a principal component analysis (PCA) to identify global differences in nectar metabolites. In general, there was little separation between the different genotypes. The strongest separation was between line #2 and the WT, while the separation between lines #1 or #5 and the WT was not as strong (Fig. 6A). A clustered heatmap of log-transformed relative abundances for all 150 metabolites revealed substantial variation in the global metabolite profiles of each genotype (Fig. 6B), without a clear clustering pattern. Interestingly, line #2 had lower relative abundance of most NCs compared to the WT (Fig. 6B), likely due to a lower amount of nectar production compared to lines #1 and #5 (Supplementary Fig. S11). Despite weak general changes to nectar metabolic profiles, a number of individual metabolites were significantly and consistently different between the transgenic lines and the WT (Table 1). Specifically, salicylic acid (SA, the identity of which was confirmed using a pure standard; Supplementary Fig. S12) was over two-fold lower in all pCRC::ADG1-amiRNA lines measured compared to the WT (Fig. 6C; Table 1). SA, which is predominantly produced from plastid-derived precursors, has been shown to be important for systemic acquired resistance to pathogens in many plant species, including *Arabidopsis* (Zhang et al. 2013). SA thus might play a similar protective role in nectaries during nectar secretion. Based on this result, we searched for other NCs related to fungal or bacterial pathogens. We identified another significantly different NC between the transgenic lines and the WT, neohydroxyaspergillic acid (NHAA), which is related to aspergillic acid, a compound that is produced by the common plant pathogen yellow-green mold (*Aspergillus flavus*) (Lebar et al. 2019). The level of NHAA in the nectar of pCRC::ADG1-amiRNA mutants was over 2-fold higher than in the WT (Table 1), suggesting that the nectar might be colonized by this specific fungus. In addition to these NCs potentially associated with biotic stress, we detected a number of putatively identified other NCs that were consistently different between pCRC::ADG1-amiRNA lines and the WT (Table 1). This suggests alternative functions of nectary starch besides the production and secretion of nectar sugars, which will provide many interesting directions of research for future studies.

**Table 1.** List of significantly different compounds from *Arabidopsis thaliana* mutants defective in starch synthesis (pCRC::ADG1-amiRNA) compared to WT

Mode	Name	m/z	RT	Log2FC					
				(amiR1/WT)		(amiR2/WT)		(amiR5/WT)	
−	Crotetamide	225.16	1.74	−2.52	*	−3.04	*	−3.23	**
−	Salicylic acid	137.02	0.65	−2.29	****	−1.44	***	−1.04	**
+	3-Amino-4-hydroxybenzoate	195.08	1.97	−1.59	**	−1.34	**	−1.02	*
−	2,4-Dinitrophenol	183.00	0.58	−1.07	**	−0.80	*	−1.06	**
+	Methyl palmitate	288.29	1.25	−0.79	**	−0.99	***	−0.71	**
+	*N*-Alpha-acetyllysine	189.12	3.60	−0.59	**	−0.61	**	−0.57	**
−	D-(+)-Glucose	215.03	3.74	0.06	n.s.	−0.86	****	−0.11	n.s.
−	P-Octopamine	152.07	0.89	0.40	*	0.91	***	1.28	**
−	*N*-Acetylleucine	172.10	1.05	0.59	**	0.87	***	1.18	****
+	(2-Butyl-1*H*-imidazol-4-yl)methanol	155.12	1.22	0.87	**	1.75	**	1.83	****
−	4-[3-(Dimethylamino)propylamino]phenol	193.13	0.94	1.29	**	2.27	***	2.80	****
−	Neohydroxyaspergillic acid	239.14	0.74	1.45	**	3.02	***	2.86	****
−	(3S)-3-[(2S)-2-Amino-3-oxobutyl]-2-pyrrolidinone	169.10	1.00	1.49	**	2.46	****	2.89	****

Relative differences are represented as Log-2 fold change (Log2FC), which was calculated for average abundance of each mutant (amiR1 = line #1, amiR2 = line #2, amiR5 = line #5) compared to WT. Statistics were conducted comparing the abundances of mutant and WT (two-sample *t*-test; *N* = 8; *P < 0.1, ** *P* < 0.05, *** *P* < 0.005, ****P < 0.0005).

### Tobacco starchless mutants have no change in nectar production and secretion

To understand whether the role of nectary starch in nectar production is conserved, we next examined nectar secretion in a woodland tobacco (*Nicotiana sylvestris*) mutant defective in plastidial phosphoglucomutase (*pgm*; Hanson and McHale 1988). We chose *N. sylvestris* since the *pgm* mutant was readily available and because *Nicotiana* spp. have frequently been the focus of nectar-related studies that have hypothesized a connection between nectary starch degradation and nectar sugar production (Ren et al. 2007; Liu and Thornburg 2012; Tiedge and Lohaus 2017). Similar to *Arabidopsis pgm1*, the *N. sylvestris pgm* is unable to produce starch in its leaves (Zeeman and Solhaug 2022). We examined the accumulation of nectary starch in WT *N. sylvestris* flowers at two developmental stages, as described previously (Ren et al. 2007). Briefly, S9 represents a closed flower (no nectar), and S12 represents an open flower (nectar present; Supplementary Fig. S13A). In S9, nectary starch is present, which is then degraded as the flower opens, with S12 nectaries containing little, if any, starch (Supplementary Fig. S13B). Similar to *pgm1* mutants in *Arabidopsis*, *pgm* mutants of *N. sylvestris* had reduced accumulation of nectary starch at S9 and S12 (Supplementary Fig. S13B). In contrast to the hexose-dominant nectar of *Arabidopsis*, *N. sylvestris* produces a nectar that has equimolar amounts of Suc and hexoses (Tiedge and Lohaus 2017). There was no change in the concentration of Suc or Glc in the nectar, nor in nectar volume, in *N. sylvestris pgm* mutants compared to the WT (Supplementary Fig. S13, C and D). This suggests that, like in *Arabidopsis*, floral nectar production in *N. sylvestris* does not depend on nectary starch.

## Discussion

Here, we provide evidence supporting the hypothesis that floral nectar can be produced without prior storage of carbohydrate as nectary starch. First, despite the coincidence of nectary starch degradation, flower opening, and nectar production (Figs. 1 and 2), diel variation in nectar production and secretion did not align with the degradation of nectary starch reserves in WT open flowers (Fig. 2). Second, mutants defective in both whole-plant and nectary-specific starch synthesis exhibited largely inconsistent changes in nectar Glc (Figs. 3 and 4). Mutants that were unable to allosterically regulate AGPase produced less nectar Glc, suggesting that nectary starch synthesis might compete with nectar sugars for phloem-derived C (Fig. 5). Finally, nectar metabolomics experiments with nectary-specific starch-deficient mutants revealed multiple nonsugar NCs possibly connected to nectary starch degradation (Fig. 6). Based on our results, we conclude that nectary starch could provide carbon for the production of NCs associated with biotic stress, which will be discussed further below.

### Diel changes in nectar sugar suggest importance of nectar recovery via resorption

Our results supported a dynamic daily secretion model involving the induction of *SWEET9* and *CWINV4* expression at dawn (ZT0), leading to nectar secretion in open (S14/S15) flowers (Supplementary Fig. S3, Fig. 2C). This active secretion in the morning is followed by a decrease in nectar Glc in the afternoon and evening (Fig. 2C). Since the plants were not exposed to pollinators, the decrease in nectar Glc in the afternoon and evening could represent a resorption and recovery of nectar sugars. Resorption could also occur during the later developmental stages of the flower, as nectar Glc was also lower in post-secretory stage (S16) compared to secretory flowers (S14/S15; Fig. 1B).

Resorption of nectar sugars has been observed in other species, including *C. pepo* (Cucurbitaceae; Nepi et al. 2001; Solhaug et al. 2019a), Himalayan cherry (*Cerasus cerasoides*; Rosaceae; Dong et al. 2016), and greater butterfly orchid (*Platanthera chlorantha*; Orchidaceae; Stpiczyńska 2003). In *C. pepo*, upon addition of labeled ^14^C-Suc to the nectar of male and female flowers, ^14^C was observed in nectaries and vascular tissues (Nepi and Stpiczyńska 2007). In pollinated female flowers, the highest amount of ^14^C label was observed in developing ovules relative to other tissues, suggesting that successful pollination may initiate nectar resorption to provide carbon for developing seeds (Nepi and Stpiczyńska 2007). Additionally, when researchers removed floral nectar (without increasing pollination) from winter tree orchid (*Mystacidium venosum*; Orchidaceae) and pinto penstemon (*Penstemon roseus*; Plantaginaceae) flowers, lower fruit and seed yield was observed (Luyt and Johnson 2002; Ornelas and Lara 2009). Together, these data suggest that resorbed and recovered nectar sugars (or other compounds) might contribute substantially to plant carbon balance and reproductive output. However, another possible explanation for our results is that the nectar Glc was converted to some other metabolite in the nectar throughout the day (i.e. oligosaccharides or some other temporary storage form).

### Nectar sugar production is not dependent on nectary starch

Although a temporal coincidence between nectary starch degradation and nectar sugars has been reported in numerous studies (Ren et al. 2007; Liu and Thornburg 2012; Lin et al. 2014; Solhaug et al. 2019a), there was no consistent change in nectar Glc accumulation during any stage (S13, S14/15) or any time of day (ZT0, ZT6) in whole-plant (*adg1*, *pgm1*) or nectary-specific (pCRC::ADG1-amiRNA) mutants that were disrupted in starch synthesis (Figs. 3 and 4). Thus, our data suggest that nectar sugar production and secretion do not strictly depend on nectary starch reserves.

Surprisingly, nectary-specific expression of *GlgC-TM*, encoding a misregulated version of AGPase, led to inconsistent changes in nectary starch (Fig. 5). Interestingly, the overaccumulation of nectary starch (or changes in starch-related metabolites) may have initiated endogenous feedback mechanisms to transcriptionally suppress starch synthesis in flowers, as we also observed consistently lower floral *ADG1* expression in each pCRC::GlgC-TM line measured (Supplementary Fig. S9A). Similar feedback inhibition mechanisms targeting GlgC-TM (i.e. translation inhibition) might explain why four of the five lines did not produce strong starch excess phenotypes, despite high *GlgC-TM* transcript levels (Fig. 5, A and B).

Despite the variable starch excess phenotypes for independent lines, pCRC::GlgC-TM mutants had consistently lower nectar Glc compared to the WT (Fig. 5C). This reduced nectar phenotype might have been due to increased starch synthesis in pCRC::GlgC-TM lines, which competed for phloem-derived carbon with the nectar secretion pathway involving SPS1F/2F, SWEET9, and CWINV4. Although there was not a consistent increase in nectary starch in the pCRC::GlgC-TM lines (Fig. 5, A and B), GlgC-TM possibly diverted carbon away from nectar through the synthesis and accumulation of ADP-Glc or malto-oligosaccharides, rather than the production of Suc precursor UDP-Glc.

Unfortunately, our ability to meaningfully detect small changes in nectary starch is negatively influenced by the lack of true nectary starch quantitation with enzymatic assays. Enzymatic assays are not feasible in the *Arabidopsis* system, as the flowers are simply too small to conduct suitably replicated and rigorous experiments on nectaries specifically. Our image-based quantitation of nectary starch was a useful and important tool to enhance our data and provided a way for us to deepen our analyses, but this frequently used approach suffers from various biases and is therefore not ideal. Like in many sub-foci of plant biology, *Arabidopsis* provides an initial genetic basis that allows us to build our understanding and generate hypotheses to inform future transcriptomic, proteomic, or metabolomic studies in species with larger nectaries, such as *C. pepo* (Solhaug et al. 2019a), *Nicotiana* spp. (Liu and Thornburg 2012), or *B. rapa* (Minami et al. 2021), where nectary-specific quantitation of genes, proteins, or metabolites (including starch) would be more feasible.

*Arabidopsis* and *N. sylvestris* are both self-pollinating, and starchless mutants in both species do not have consistent reductions in nectar production (Figs. 3 and 4, Supplementary Fig. S13), supporting conserved starch-independent secretion and a prominent role for phloem-derived carbon in the production of nectar sugars. Despite this, nectary starch might provide an emergency “fail-safe” to ensure that some nectar is produced even when plants experience unexpected stresses, many of which (such as drought) can influence the supply of phloem-derived photosynthate from the leaves (Hu et al. 2023). This may be particularly relevant for species that rely on outcrossing and should be tested in future studies including obligate outcrossers. Together, our data support a prominent and conserved role for phloem-derived carbon, rather than nectary starch, in the production of nectar sugars.

### Transcriptional regulation of nectary carbon metabolism

While defects in nectary starch did not have consistent impacts on nectar sugar production, expression of nectary-enriched genes varied consistently, which suggested possible reconfigurations of nectary carbon metabolism. *CWINV4* expression was consistently lower in all pCRC::ADG1-amiRNA lines measured, even those that did not have lower nectar sugar, indicating that specific repression of nectary starch synthesis influences the expression of this gene (Supplementary Fig. S7). CWINV4 activity is probably important for the import of phloem-derived sugar into nectaries, as nectarless *sweet9* and *sps1f/2f* mutants both showed an overaccumulation of nectary starch (Lin et al. 2014), while nectarless *cwinv4* mutants did not (Ruhlmann et al. 2010), suggesting a blockage of phloem sugar uptake into nectaries. In our study, *SWEET9* expression appeared closely tied to the level of sugar in the nectar. The pCRC::ADG1-amiRNA mutants that had the lowest nectar Glc also had the lowest expression of *SWEET9*, while those mutants that did not have altered nectar Glc did not have reduced *SWEET9* (Fig. 4; Supplementary Fig. S7). Lin and others found that *Arabidopsis* pSWEET9::SWEET9-eGFP lines possessing higher SWEET9 in nectaries had two-fold higher nectar Glc compared to WT (Lin et al. 2014). This result suggests that, under standard conditions, nectar sugars are limited by the ability of SWEET9 to export Suc.

Due to the small size of *Arabidopsis* flowers, we used whole flowers in our gene expression analyses of nectary-enriched genes. However, the genes that we focused on in our expression analysis are either specific to nectaries (*CWINV4* and *SWEET9*) or highly enriched in nectaries (*SPS2F*) relative to nonnectary reference tissues, according to previous transcriptomic data (Kram et al. 2009). These nectary-specific expression patterns gave us confidence in our conclusions regarding potential metabolic explanations of our observed variation in nectar sugars between starchless genotypes. Even still, it must be noted that whole-flower gene expression experiments should be accompanied by additional experimentation, such as mutational studies or spatial transcriptomics, before drawing any mechanistic conclusions about nectar production.

### Specialized metabolism in *Arabidopsis* nectar

Although our experiments suggest that nectary starch is not the main source of nectar sugars, nectary starch accumulation and degradation have been commonly observed in nectaries of many species (Y. Ben Peng et al. 2004; Ren et al. 2007; Solhaug et al. 2019a), and there are numerous other NCs that could be potentially derived from nectary starch. Intriguingly, SA was low in our pCRC::ADG1-amiRNA lines relative to WT (Fig. 6C). SA plays a well-known role in the defense of plant tissues against pathogenic fungi or bacteria (Klessig et al. 2018), but little is known about the connection between SA and floral nectar production. Nectary starch as a source for SA production makes sense from a subcellular perspective, as the first step of SA biosynthesis from precursor chorismate occurs in plastids (Peng et al. 2021).

Numerous studies have shown putative evidence that specialized compounds produced in nectar defend the nectar against pathogens. Specifically, a predominant nectar-derived peptide in *B. rapa* is a lipid transfer protein, called BrLTP2.1, which also was shown to have antimicrobial activity against common plant pathogens (Schmitt et al. 2018). Pathogens can easily enter plants via transfer from visiting pollinators, and sugar-rich nectar is a fertile growth medium if left undefended. For example, the fire blight bacterium (*Erwinia amylovora*) can infect floral nectaries of apple (*Malus x domestica*; Zeng et al. 2021) and common pear (*Pyrus communis*; Fotirić Akšić et al. 2023). Interestingly, variation in susceptibility for different *P. communis* cultivars can at least be partially explained by changes in the chemical constituents of the nectar, including minor sugars and polyphenols (Fotirić Akšić et al. 2023).

One limitation of our untargeted metabolomics approach is that many of the identifications are based solely on the *m*/*z* ratio and RT (Table 1), with a degree of uncertainty. Running individual standards for all NCs was not feasible, but additional targeted experiments with a SA standard supported our initial identification of SA (Supplementary Fig. S12). Other metabolites identified (e.g. NHAA) will need to be similarly retested before proceeding with future experiments. Nevertheless, our experiments identified numerous differentially accumulated NCs in pCRC::ADG1-amiRNA plants, which may be starch dependent. Together, our data suggest that NCs derived from starch could play important roles in synchronizing nectary defense responses, protecting the nectary or flower during times of stress.

## Conclusions

In summary, we have provided numerous pieces of evidence supporting a starch-independent model of nectar sugar production and secretion. In WT plants, while nectar sugar levels are developmentally linked to nectary starch degradation, nectar sugars also fluctuate diurnally without corresponding changes in nectary starch. Whole-plant mutants defective in starch synthesis did not show consistent reductions in nectary starch, while targeted removal of nectary starch led to inconsistent nectar sugar phenotypes in independent transgenics. In fact, increasing starch in nectaries actually led to decreased nectar sugar. Despite no change to nectar sugars, nectary starchless mutants had altered accumulation of other NCs, specifically those related to pathogen resistance, suggesting these compounds might be derived from carbon produced by starch degradation within the nectary. Future identification of differentially accumulated metabolites will provide many avenues for research, further improving our understanding of the role of nectary starch in nectar production and plant–pollinator interactions.

## Materials and methods

### Plant material and growth conditions

Plants were grown in climate-controlled growth chambers under long-day (16 h light/8 h dark) conditions. Light intensity was an average of 80 ± 19 µmol m^−2^ s^−1^, measured at different points in growth trays. Temperature was 21 °C during the day and 19 °C during the night, while relative humidity (RH) was 45% during the day and 55% during the night. Plants were arranged in a random order prior to bolting to minimize differences in light and water supply. Nectar was collected from plants that were within the first two weeks of bolting (between 28 and 42 days old). The *adg1-1* mutants (Wang et al. 1998) and *pgm1-1* mutants (Periappuram et al. 2000) were obtained from the Zeeman Group at ETH Zurich, while the pCRC:ADG1-amiRNA and pCRC::GlgC-TM mutants were generated in a Columbia-0 (Col-0) WT background by floral dip transformation using *Agrobacterium tumefaciens* (Clough and Bent 1998).

### Molecular cloning and vectors used

Constructs were generated using the Gateway system (Invitrogen). For nectary-specific silencing of ADG1, entry vectors were first constructed containing 3.8 kb of the *CRC* promoter region (pDONR-P4P1r-pCRC), synthesized from Col-0 genomic DNA, and a 400-bp artificial microRNA (amiRNA) targeting *ADG1* (At5g48300), synthesized using IDT Gene-Blocks (Integrated DNA Technologies), based on a modified miR319a precursor (Ossowski et al. 2008). This amiRNA was used to generate the second entry vector, pDONR221-ADG1-amiRNA. Both entry vectors were combined into a destination vector, pB7m24GW to generate the final binary vector for insertion into the WT genome (pCRC::ADG1-amiRNA). For expression of *GlgC-TM* in nectaries, pDONR-P4P1r-pCRC was combined with an entry vector containing the *GlgC-TM* sequence from *E. coli* and the 3′ UTR and chloroplast targeting sequence of Arabidopsis *ADG1* (pDONR221-GlgC-TM). As before, both entry vectors were combined with destination vector pB7m24GW to generate the final binary vector for insertion into the WT *Arabidopsis* genome (pCRC::GlgC-TM). Cloning and sequencing primers are included in Supplementary Table S2 and vector sequences are included in Supplementary Folder S1.

### Nectar collection

For our *Arabidopsis* experiments, nectar was collected with paper wicks made from Whatman No 1 (2.5 cm) cellulose filter paper (Whatman International Ltd). Since the nectar was collected on paper wicks, nectar Glc and other NCs were analyzed as total abundance per flower, as we were unable to measure volume or concentration due to the small size of the *Arabidopsis* flowers. Unless otherwise stated, floral nectar was collected between ZT3 and ZT5 in all experiments. Each biological replicate was a pooled sample of nectar collected from seven flowers, from seven plants (one flower/plant), on one wick. Nectar samples were stored at −20 °C before analysis of nectar Glc. Each wick was then placed in a 1.5-mL tube containing 500 µL of Milli-Q water, and centrifuged at 5,000 × *g* for 1 min to ensure that the wick was completely immersed in the water. The tubes were vortexed briefly, then the sugar solutions were used directly in the assay for Glc.

### Glc assay in *Arabidopsis* nectar

We used Glc as a proxy for total *Arabidopsis* nectar sugar, rather than Fru or Suc, as previous data have shown that *Arabidopsis* flowers contain little to no Suc, and the ratio of Glc:Fru in *Arabidopsis* nectar is around 1.0 (Davis et al. 1998). This Glc assay is simple, consisting of a one-step reaction, and thus is more scalable than alternative quantitative methods (i.e. LC-MS). The Glc assay was performed as described previously, with some modifications (Solhaug et al. 2019a). Briefly, 50 µL of nectar solution was added to 25 µL of Milli-Q water and 25 *µ*L of Glc assay solution, which contained 0.1 U Glc oxidase, 0.1 U horseradish peroxidase, 400 µM Ampliflu Red (stock solution dissolved in dimethyl sulfoxide), and 34 mm sodium phosphate buffer pH 7.4. All reactions were incubated for 25 min in darkness at 22 °C, then absorbance at 570 nm (A570) was measured with a plate reader, and compared to a standard curve of Glc (25–500 µM). For each nectar biological replicate, we performed two technical replicates to minimize variation caused by technical issues. Total nanomoles (nmol) of Glc per flower (T) was calculated for each sample from the concentration of the Glc in the tube (C_t_), the volume of DI water used to suspend the wick (V), and the number of flowers collected from (F). The equation, T = (C_t_ * V)/F, was then used to calculate nmol Glc/flower.

### Suc assay in tobacco nectar

For our experiments involving *N. sylvestris*, we collected nectar using microcapillary tubes that allowed us to quantify the volume (one flower/biological replicate). For nectar Suc quantification, we pretreated the samples with invertase, as described previously (Solhaug et al. 2019a), followed by quantification of Glc as described above. Samples treated with invertase were compared to Glc controls (i.e. no invertase) to determine the [Suc] in the nectar. We ran standard curves of Glc and Suc (25–500 *µ*M) to ensure that the Suc hydrolysis step was proceeding correctly.

### Starch stains in flowers

Starch was examined in floral tissues as described previously, with minor modifications (Hedhly et al. 2018). Flowers were collected at specified stages (S12, S13, S14) and placed in Carnoy's fixative solution (3:1 ethanol:acetic acid). Samples were incubated at 4 °C overnight (16–24 h); then each sample was removed and placed in 70% (v/v) ethanol for >24 h before staining for starch. Unless otherwise stated, aqueous solutions referenced as a percentage throughout the rest of this paper are v/v. The combined clear-stain solution was Herr's IKI-4^1^/_2_, as described in detail by Hedhly et al. (2018). Samples were cleared overnight (16–24 h); then patterns of starch accumulation were visualized using a Leica S9i stereomicroscope in transmission light mode.

### Image-based nectary starch content quantification

To estimate quantitative differences in nectary starch content between starch stain images, nectary boundaries were first manually defined using custom software (https://github.com/yatengLG/ISAT_with_segment_anything). A representative example of our nectary annotation is included in Supplementary Fig. S1. Images were converted to grayscale with inverted color representation to fulfill the intuition that darker staining indicates higher starch content. The pixel value within both nectaries was averaged to generate each biological replicate. All microscope and camera settings were the same for all images taken, so we used raw pixel values in our analysis. Values were compared between samples collected and measured together, as we observed slight variation in the level of WT staining between (but not within) experiments. The code is available at https://github.com/Alias-z/NectarStarch.

### Leaf starch analysis

Leaf starch was determined as described previously (Smith and Zeeman 2006), with some modifications. Leaf samples (one leaf/biological replicate) were first treated with 70% (v/v) ethanol to remove interfering soluble sugars (i.e. Suc and Glc), a method that has been used previously (Martins et al. 2013). Briefly, leaf tissue (leaf #6, 30–60 mg) was ground in 200 µL of 70% (v/v) ethanol and then heated for 5 min at 80 °C in a heating block. Samples were mixed, then another 200 µL of 70% (v/v) ethanol was added before centrifugation at 1800 × *g* for 10 min. The supernatant was discarded, and then the pellet was resuspended in 400 µL 70% (v/v) ethanol and centrifuged again at 1800 × *g* for 10 min. The supernatant was discarded again, and then the pellet (containing starch) was resuspended in 200 µL of deionized (DI) water. Each sample was divided in half, placed in separate screw-cap tubes (one control and one digest), and then incubated for 10 min at 100 °C to solubilize the starch. Samples were cooled for 5 min at room temperature (∼22 °C), before beginning digestion. Digest solution consisted of (in 100 µL) 95 µL 80 mm sodium acetate buffer, pH 4.8; 4.5 µL amyloglucosidase (∼0.3 units; Roche); and 0.5 µL of α-amylase (∼5 units; Roche). Undigested controls contained 95 µL of sodium acetate buffer (pH 4.8) and 5 µL of DI water. Control and digest solutions were added to separate samples, and then all samples were incubated at 37 °C for 4 h. Glc liberated from starch was measured similar to nectar Glc. After incubation, each starch sample was vortexed and then centrifuged for 1 min at 5,000 × *g* to remove particulate matter. A 1:10 dilution was made of the digested starch samples, and then Glc was assayed in a total volume of 100 µL, with 25 µL of starch sample, 25 µL of DI water, 25 µL of 150 mm sodium phosphate buffer (pH 7.4), and 25 µL of Glc assay solution (prepared the same as nectar Glc assay). After 25 min in the dark, A570 was measured with a spectrophotometer and compared to a standard curve of D-Glc, as with the nectar sugar assay. For each biological replicate, we performed two technical replicates in the sugar assay to minimize variation caused by technical issues.

### Analysis of gene expression with RT-qPCR

Flowers and leaves were collected and flash-frozen in liquid nitrogen. Unless otherwise stated, each biological replicate of flower tissue was from seven flowers taken from four separate plants (two flowers from each of three plants, one flower from the fourth), whereas for leaves each biological replicate was one leaf taken from separate plants. Flowers were taken from plants at mid-day (ZT6–ZT8). RNA was extracted with a SV total RNA isolation system (Promega), and cDNA was made from ∼500 ng of RNA using a M-MLV Reverse transcriptase reaction (Promega). Gene expression was analyzed with an Applied Biosystems SYBR Green PCR Master Mix (Thermo Fisher Scientific). Around 10 ng of cDNA was used for each RT-qPCR. RT-qPCR was performed with a LightCycler 480 II RT-qPCR machine (Roche). Relative expression was calculated using ΔCt, and *UBIQUITIN 10* (*UBQ10*) as a housekeeping gene. For each biological replicate, we performed two technical replicates to minimize variation caused by technical issues. Primer sequences are included in Supplementary Table S1.

### Sampling and extraction for LC-MS/MS

Nectar samples were collected as described above. Each sample is pooled nectar collected from seven flowers (from seven plants, one flower/plant) with a paper wick and then suspended in 500 µL of DI water. From each sample, 100 µL was taken for the nectar sugar assays described above (50 µL × 2 technical replicates), and these same samples (now 400 µL) were extracted and used for LC-MS/MS. As a blank, 500 µL of DI water was combined with a paper wick (no nectar), and then 100 µL was removed, and 400 µL of samples and blank was used in the following extraction and analysis of nectar metabolites.

### LC-MS/MS analysis of polar metabolites

We performed LC-MS/MS analysis using the Functional Genomics Center at ETH Zurich (https://fgcz.ch). Each sample (150 µL) was mixed with 600 μL of methanol and extracted on a shaker for 10 min at 10 °C at 1,000 rpm. Samples were dried down under N_2_ and reconstituted in 100 µL of ACN:H_2_O (9:1) by shaking for 10 min at 10 °C at 1,000 rpm. Samples were vortexed and centrifuged (16,000 × *g*, 4 °C, 10 min). of the supernatant, 60 µL was transferred to a glass vial (Total Recovery Vials, Waters, Milford, MA, USA) for LC-MS/MS injection. The QC pool consisted of 10 μL of each sample.

NCs were separated on a Thermo Vanquish Horizon Binary Pump UPLC (Thermo Fisher Scientific) equipped with a Waters (Eschborn, Germany) Premier BEH amide column (150 mm × 2.1 mm, 1.7 µm). Injection volume was 10 μL, and autosampler temperature was set to 5 °C. The solvent system consist of buffer A (ACN:H_2_O, 1:19) and buffer B (ACN:H_2_O, 19:1), both with 10 mm NH_4_HCO_3_ adjusted to pH 9. Separation was carried out at a flow rate of 0.4 mL min^−1^, with a constant column temperature of 40 °C. The gradient elution profile was the same for both positive and negative ionization modes (0.0 min, 1% A; 0.1 min 1% A; 6 min 70% A; 7 min 70% A; 7.5 min 1% A, 12 min 1% A). The UPLC was coupled to a Q Exactive mass spectrometer (Thermo Fisher Scientific) by a HESI source. MS data were acquired using negative and positive polarization and data-dependent acquisition (DDA, top 5). Full-scan MS spectra were acquired in profile mode from 70 to 1,050 *m*/*z* with an automatic gain control target of 1e6, an Orbitrap resolution of 70,000, and a maximum injection time of 100 ms. The 5 most intense charged (*z* = + 1 or +2) precursor ions from each full scan were selected for collision-induced dissociation fragmentation. The precursor was accumulated with an isolation window of 1 Da, an automatic gain control value of 1e5, a resolution of 17,500, a maximum injection time of 50 ms, and fragmented with a normalized collision energy of 10, 20, and 40 (arbitrary unit). Data for all measured compounds are included in Supplementary Data Set 1.

### LC-MS/MS targeted analysis of SA

NC extracts derived from WT and pCRC::ADG1-amiRNA mutant (line #2) nectar, previously used for untargeted metabolomics analysis, were dried under nitrogen and reconstituted in 60 µL of 90% (v/v) acetonitrile for targeted analysis. Samples were briefly vortexed and shacked for 10 min at 4 °C to ensure solubilization. Solubilized samples were centrifuged (16,000 × *g*, 4 °C, 10 min), and 50 µL of the supernatant was transferred to narrow-bottom glass vials (Total Recovery Vials, Waters) for LC-MS injection. Method blanks, QC standards, and pooled samples were prepared in the same way to serve as quality control for the measurements. The serial dilution of SA (Sigma Aldrich) was also transferred to the same vials prior to injection.

LC-MS analysis of metabolomics samples was performed at the Functional Genomic Center Zurich, on a Exploris 480 (Thermo Fisher Scientific) mass spectrometer coupled to a Thermo Vanquish LC (Thermo Scientific) by a HESI source. Samples were separated using a 12-min gradient with a constant flow of 0.4 mL/min on a BEH amide analytical column (ACQUITY Premier BEH amide Column, 1.7 µm, 2.1 × 150 mm) kept at 40 °C (applying a gradient of 10 mm ammonium bicarbonate in 95% water (A) and 10 mm ammonium bicarbonate in 95% acetonitrile (B) from 99% B to 30% B over 12 min) while the autosampler was kept at 4 °C. MS1 (molecular ion) and MS2 (fragment) data were acquired in negative polarization mode and full MS/dd-MS² (top 5) over a mass range of 70 to 1,050 *m*/*z*, at MS1 resolution of 70,000 and MS2 resolution >17,500. To confirm the identity of SA in nectar, the RT and the mass spectra of the two pure compounds were acquired with the same LC-MS method. Additionally, to estimate the relative abundance of SA in *Arabidopsis* nectar, serial dilutions of the pure compounds were used to generate external calibration curves.

The mass spectrometry data were handled and stored using the local laboratory information management system B-Fabric (Türker et al. 2010). The identity of SA in nectar was confirmed by comparing the extracted chromatogram for the molecular ion corresponding to SA (*m*/*z* 137.0239, mass tolerance: 5 ppm) in the pure standard and the nectar. Furthermore, the identity was confirmed at the MS2 level, by comparing the spectra of SA in nectar against the MassBank of North America database entry for SA (MoNA ID: CCMSLIB00012866788).

### Data analysis

Statistical analyses were done in Excel (for two-sample *t*-tests) or R (for one-way ANOVA and two-way ANOVAs). For data with comparing mutants to WT, we used two-sample *t*-tests to compare each mutant to WT individually. For data with multiple timepoints or developmental stages, we used a one-way or two-way ANOVA with Tukey's post hoc tests to compare each stage to each other. Untargeted metabolomics data were aligned and searched against databases with the Compound Discoverer software (Thermo Fisher Scientific). Compound identification was performed against in-house mass lists, online libraries and MS2 spectra libraries. Quality controls were run on pooled samples and reference compound mixtures to verify technical accuracy and stability. The PCA for the metabolomics data was conducted in R using the prcomp() function.

### Accession numbers

*PGM1* (At5g51820), *ADG1* (At5g48300), *SPS2F* (At5g11110), *CWINV4* (At2g36190), and *SWEET9* (At2g39060).

## Supplementary Material

kiaf515_Supplementary_Data

## Data Availability

The data underlying this article are available in the article and its online supplementary material.

## Supplementary data

The following materials are available in the online version of this article.

**Supplementary Figure S1.** Illustration of nectary starch quantification based on staining intensity.

**Supplementary Figure S2.** All pictures from diel nectary starch accumulation experiment.

**Supplementary Figure S3.** Expression of nectary-related genes in floral tissues during different times throughout a diel cycle.

**Supplementary Figure S4.** All pictures from starch synthesis mutants.

**Supplementary Figure S5.** All pictures from ADG1-amiRNA lines.

**Supplementary Figure S6.** pCRC::ADG1-amiRNA lines have similar gene silencing, no leaf phenotypes compared to WT.

**Supplementary Figure S7.** pCRC::ADG1-amiRNA lines have reduced expression of nectary-enriched genes in floral tissues.

**Supplementary Figure S8.** All pictures from pCRC::GlgC-TM lines.

**Supplementary Figure S9.** pCRC::GlgC-TM lines have similar expression of *GlgC-TM* and *ADG1* in leaves and flowers.

**Supplementary Figure S10.** Leaf starch accumulation in pCRC::GlgC-TM lines.

**Supplementary Figure S11.** Nectar sugar in pCRC::ADG1-amiRNA lines used in metabolomics experiment.

**Supplementary Figure S12.** Targeted validation of SA compound identification.

**Supplementary Figure S13.** Nectar volume and sugar concentration in WT and starchless *pgm* mutants of *N. sylvestris*.

**Supplementary Table S1.** Primer sequences used in gene expression studies.

**Supplementary Table S2.** Primer sequences used in cloning.

**Supplementary Folder S1.** Plasmid sequences used in cloning.

**Supplementary Data Set 1.** Complete list of unique metabolites detected in *Arabidopsis* with untargeted LC-MS/MS.

## Dive Curated Terms

The following phenotypic, genotypic, and functional terms are of significance to the work described in this paper:

UBQ10 Gramene: AT4G05320
UBQ10 Araport: AT4G05320

starch CHEBI: CHEBI:28017

leaf AmiGo: PO:0025034

nectary AmiGo: PO:0009035

PGM Gramene: AT5G51820
PGM Araport: AT5G51820

SWEET9 Gramene: At2g39060
SWEET9 Araport: At2g39060

ADG1 Gramene: AT5G48300
ADG1 Araport: AT5G48300

CWINV4 Gramene: At2g36190
CWINV4 Araport: At2g36190
