# Supplementary material for: Nectary starch degradation affects nectar chemical composition, but not nectar sugars, in Arabidopsis thaliana
Source: Plant Physiol. 2025 Oct 15;199(3):kiaf515. doi: 10.1093/plphys/kiaf515 (PMC12608078; doi:10.1093/plphys/kiaf515)
Supplement: kiaf515_Supplementary_Data [file kiaf515_supplementary_data.zip › SupplementaryFigs_PP2024-RA-00830R1_R3.pdf]

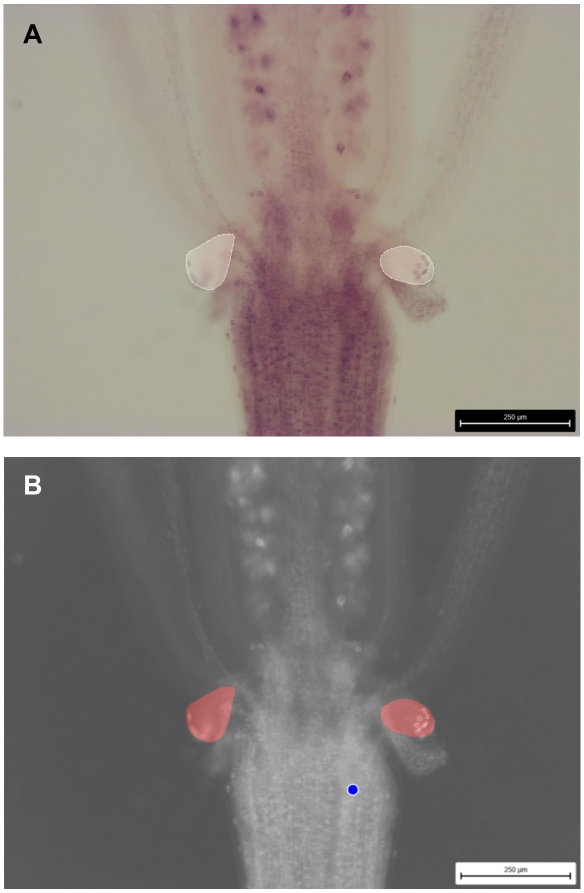

Supplementary Figure S1. Illustration of nectary starch quantification based on staining intensity. (A) Manually annotated nectary boundaries are shown as highlighted polygons. (B) Grayscale image with inverted color representation, where darker pixels indicate higher starch content. The absolute value for each nectary (highlighted in red) was calculated as the mean pixel intensity within its boundary. Scale bar = 250 μm.

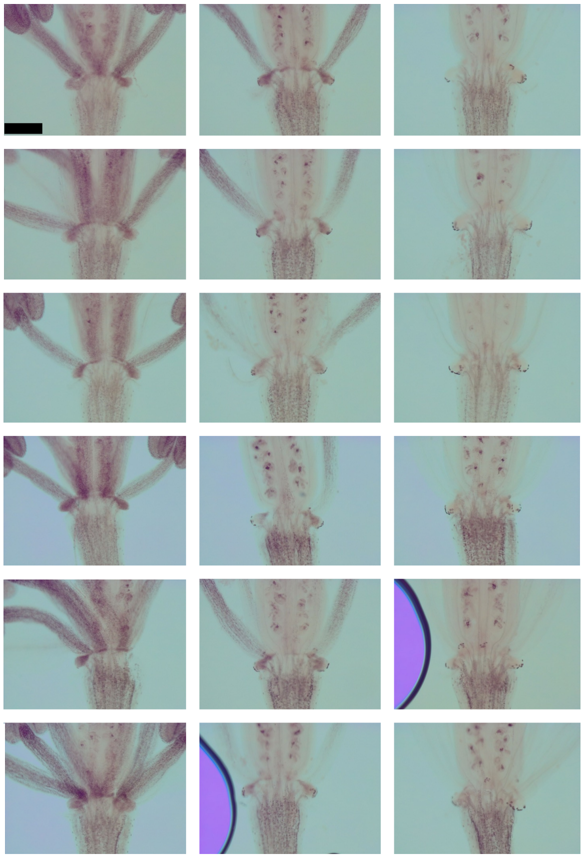

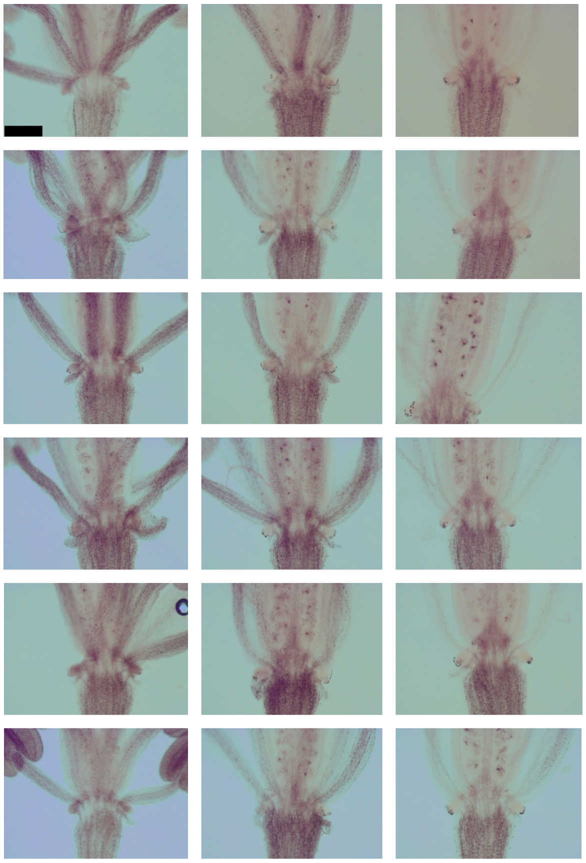

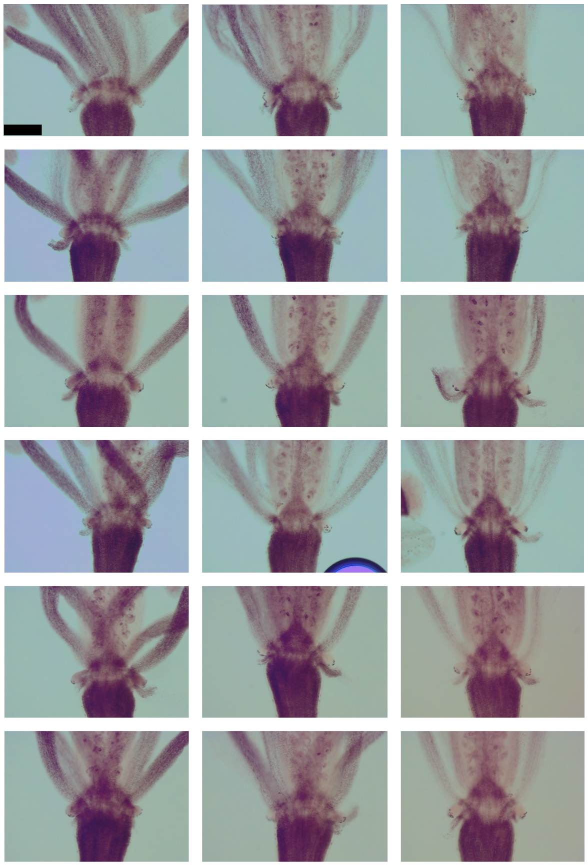

Supplementary Figure S2. All pictures from diel nectary starch accumulation experiment. Starch was measured in wild-type (WT) flowers using a combined clear-stain protocol (iodine-based) as described in methods. Flowers were harvested at the indicated times and developmental stages for the flowers (ZT = zeitgeber time, 0 = dawn; S12 = stage 12, S13 = stage 13, S14 = stage 14). Each horizontal triplicate of three flowers from three stages (S12, S13, S14) were taken from the same plant to minimize plant-to-plant variation and ensure the correct stage was collected. Scale bar = 250 µm, which applies to all eighteen images in each ZT group. These data are from one experiment.

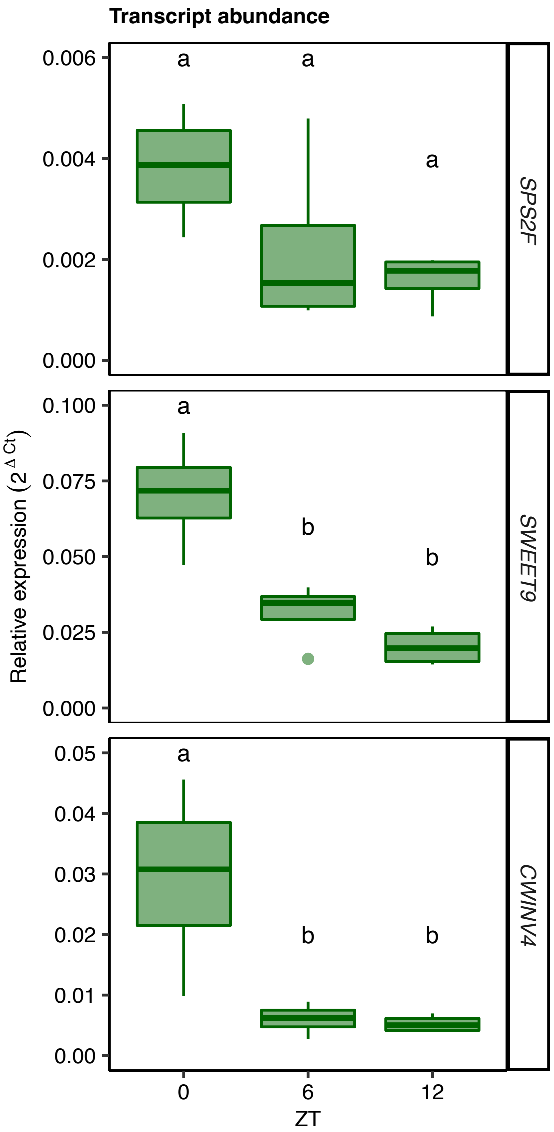

Supplementary Figure S3. Expression of nectary-related genes in floral tissues during different times throughout a diel cycle. Gene expression was measured with RT-qPCR in wild-type (WT) flowers as described in methods. Transcript abundance was measured as the number of cycles to reach a threshold fluorescence (Ct), was normalized to housekeeping gene *UBQ10*, and has been linearized for this figure ($2^{\Delta Ct}$). Each point represents pooled RNA of seven flowers taken from three different plants (two flowers from each of two plants, three flowers from the third) at three times throughout the day (ZT = zeitgeber time, ZT0 = dawn). Boxes that share a letter are not significantly different from one another (N=4 biological replicates; One-way ANOVA with Tukey post-hoc test, $p<0.05$). The center line within each box is the median (Q2), while the box represents the interquartile range (IQR), extending from the first quartile (Q1) to the third quartile (Q3), and the whiskers represent 1.5x the IQR from Q1 and Q3. Outliers are shown as individual points. These data are from one experiment.

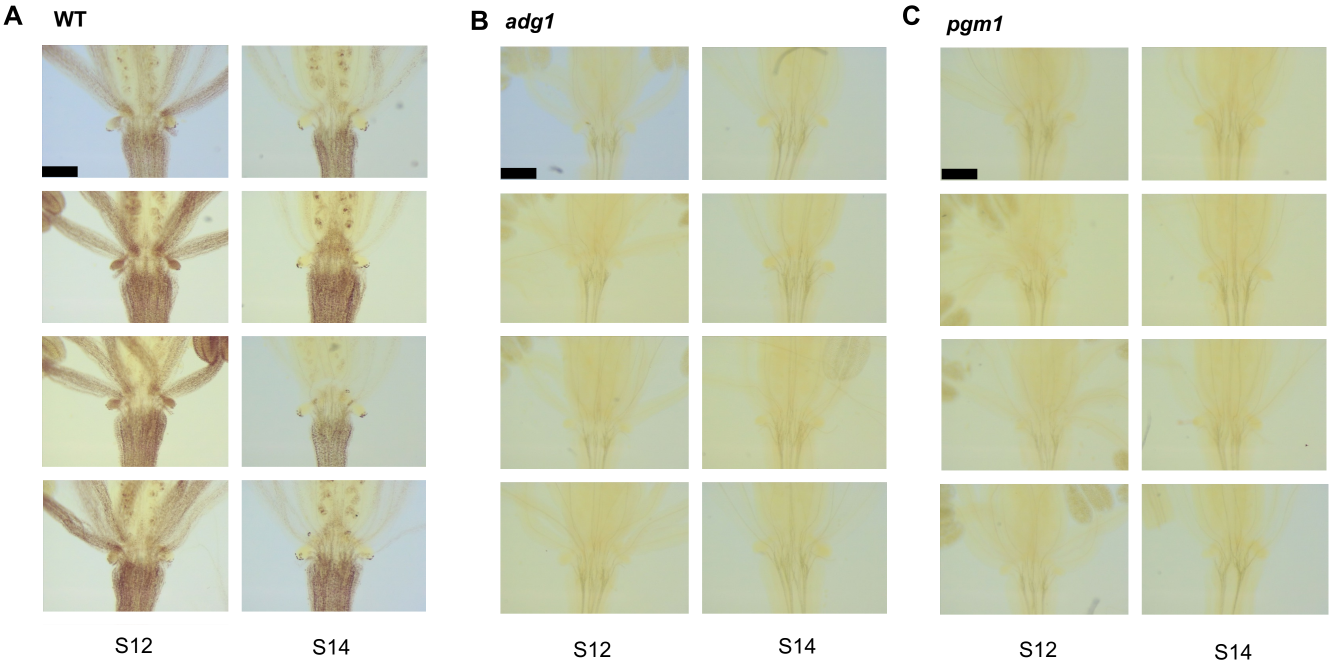

Supplementary Figure S4. All pictures from starch synthesis mutants. (A-B) Flower starch stain of (A) wild-type (WT), (B) *adg1* and (C) *pgm1* mutants. Floral starch was measured using a combined clear-stain protocol (iodine-based) as described in methods. Flowers were harvested at zeitgeber time 6 (ZT6), at indicated developmental stages for the flowers (S12 = stage 12, S14 = stage 14). Each horizontal set of flowers from different stages (S12, S14) were taken from the same plant to minimize plant-to-plant variation and ensure the correct stage was collected. Scale bar = 250 μm, which applies to all eight images in each genotype group. These data are from one experiment.

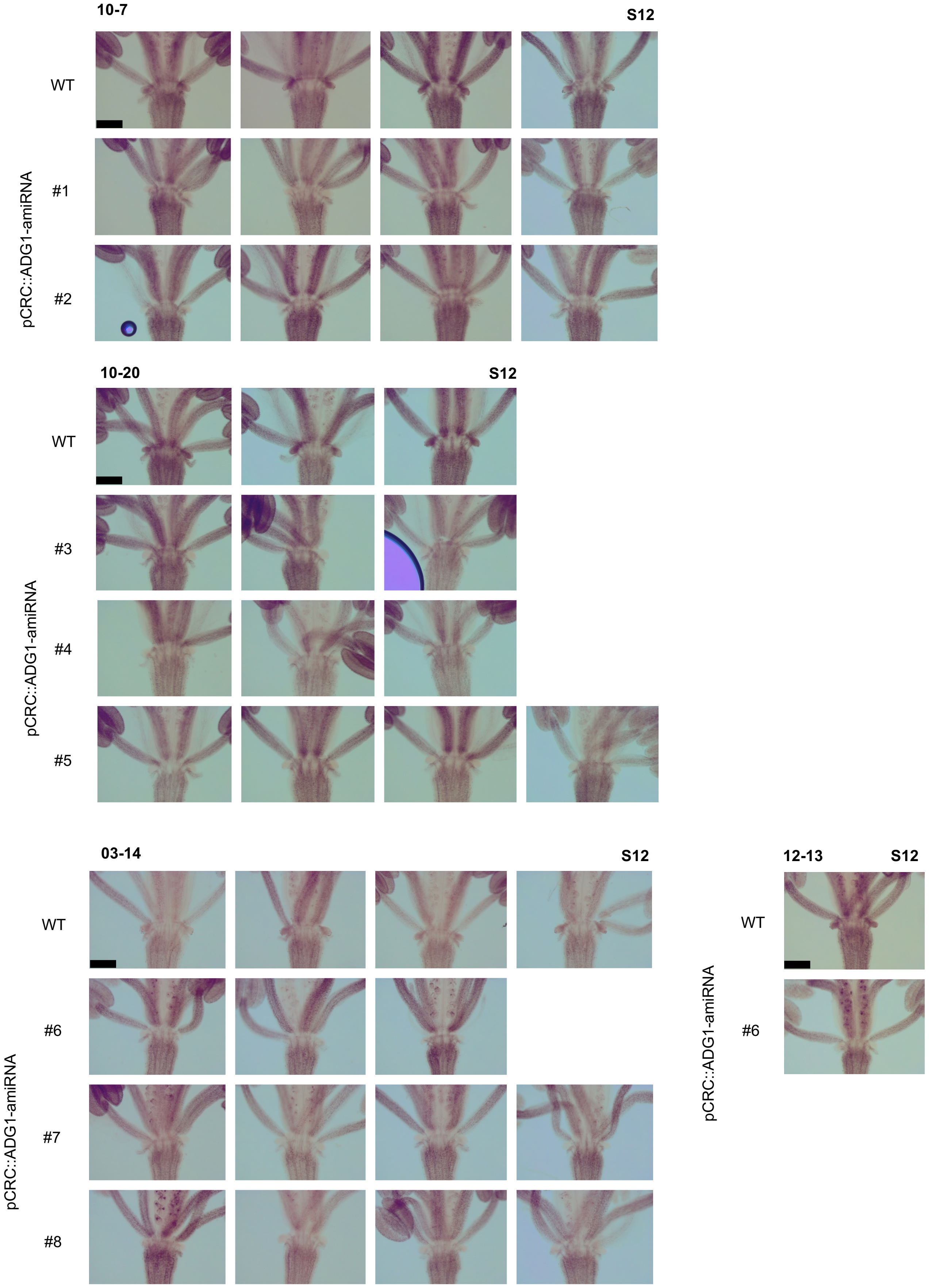

Supplementary Figure S5. All pictures from ADG1-amiRNA lines. Flower starch stain of wild-type (WT) and eight independent pCRC::ADG1-amiRNA lines. Floral starch was measured using a combined clear-stain protocol (iodine-based) as described in methods. Flowers were harvested at zeitgeber time 6 (ZT6) and at floral stage 12 (S12). Each flower was taken from a different plant. These data are from four experiments, and each group of images includes the date of harvest and starch analysis, and images were analyzed and compared to the WT that was analyzed on the same day. Each horizontal set of flowers from different lines were taken from the same plant. Scale bar = 250 μm, which applies to all images within each date group.

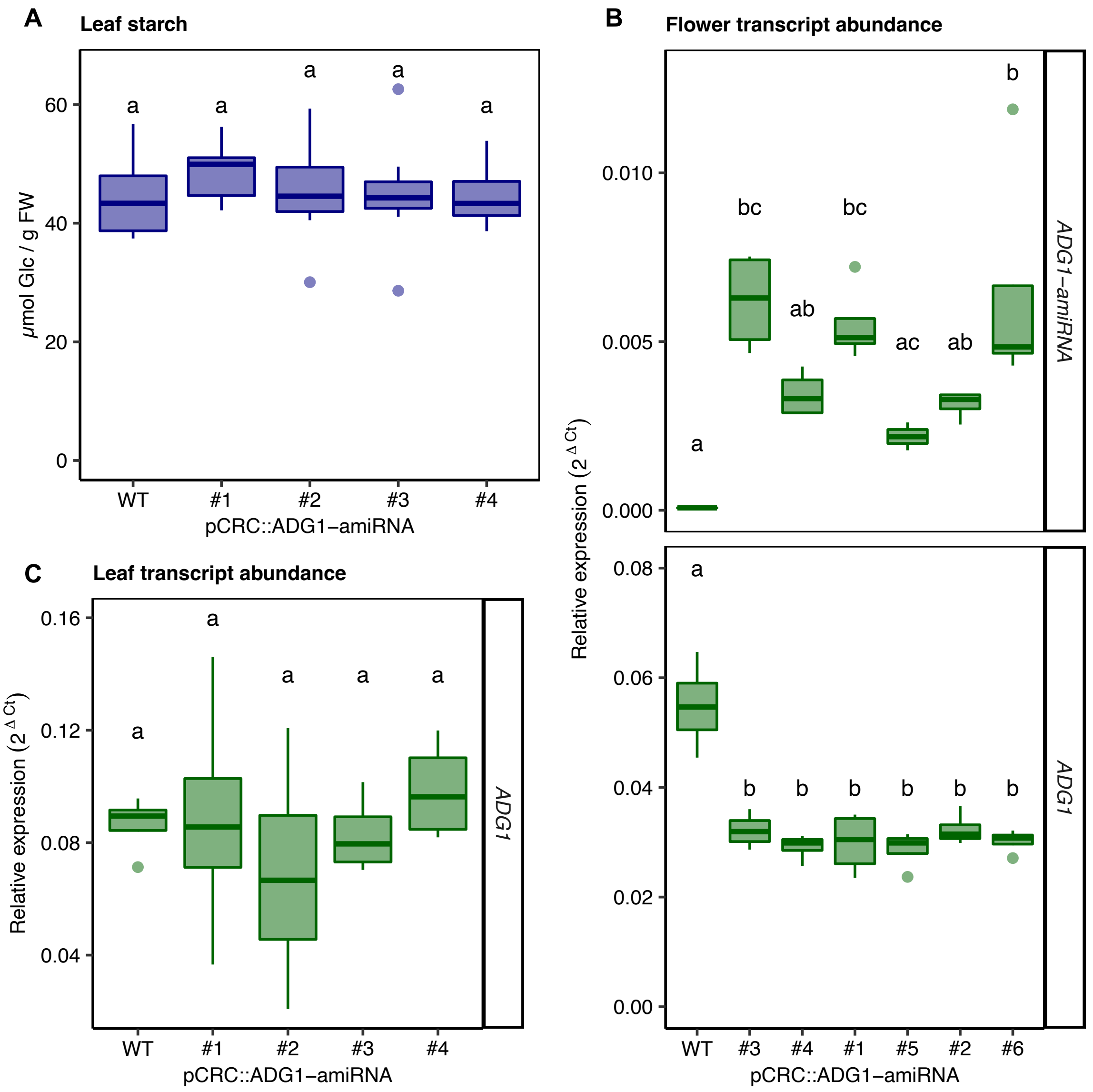

Supplementary Figure S6. pCRC::ADG1-amiRNA lines have similar gene silencing, no leaf phenotypes compared to wild-type (WT). (A) Leaf starch, in micromoles of glucose per gram fresh weight (μmol Glc / g FW), taken from leaf 6 of 22-day old plants and measured enzymatically as described in methods. (B-C) Analysis of (B) flower and (C) leaf transcript abundance for pCRC::ADG1-amiRNA lines compared to WT. Transcript abundance was measured as the number of cycles to reach a threshold fluorescence (Ct) and has been linearized for this figure ($2^{\Delta Ct}$). *ADG1-amiRNA* transcript was measured with stem-loop RT-qPCR (normalized to *sno85-miRNA*), while *ADG1* transcript was measured with normal RT-qPCR (normalized to *UBQ10*), both of which are described in methods. Each biological replicate represents pooled RNA of seven flowers taken from three different plants (two flowers from each of two plants, three flowers from the third). Boxes that share a letter are not significantly different from one another (One-way ANOVA with Tukey post-hoc test, for A, N=7 biological replicates, while N=4 biological replicates for B and C; $p<0.05$). The center line within each box is the median (Q2), while the box represents the interquartile range (IQR), extending from the first quartile (Q1) to the third quartile (Q3), and the whiskers represent 1.5x the IQR from Q1 and Q3. Outliers are shown as individual points. These data are each from one experiment.

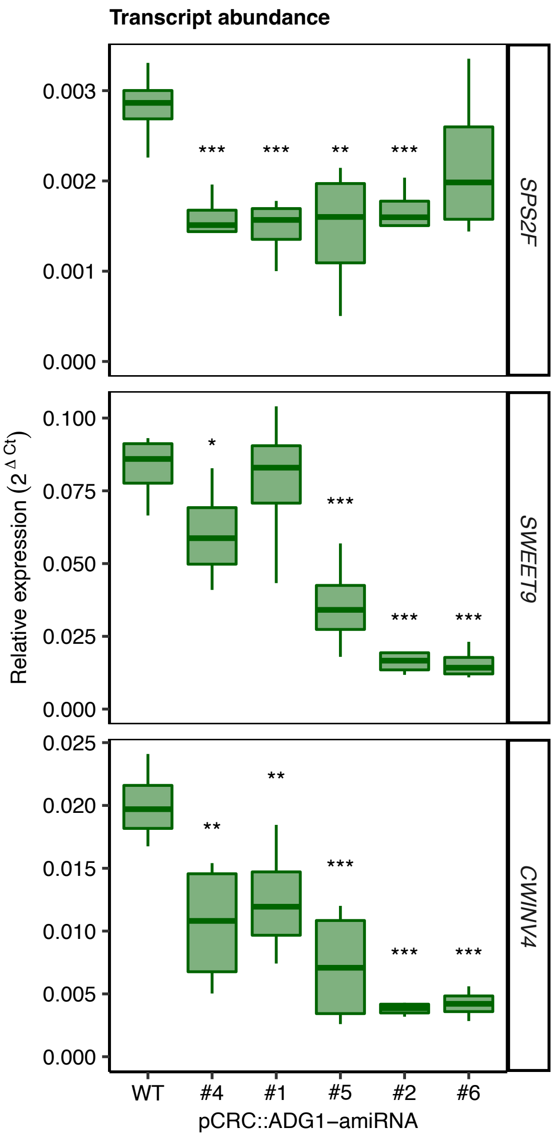

Supplementary Figure S7. pCRC::ADG1-amiRNA lines have reduced expression of nectary-enriched genes in floral tissues. Gene expression was measured with RT-qPCR in wild-type (WT) and pCRC::ADG1-amiRNA mutants as described in methods. Transcript abundance was measured as the number of cycles to reach a threshold fluorescence (Ct), was normalized to housekeeping gene *UBQ10*, and has been linearized for this figure ($2^{\Delta Ct}$). Each point represents pooled RNA of seven flowers taken from three different plants (two flowers from each of two plants, three flowers from the third). N=3 biological replicates; 2-sample t-test, *=p<0.1, **=p<0.05, ***=p<0.005. The center line within each box is the median (Q2), while the box represents the interquartile range (IQR), extending from the first quartile (Q1) to the third quartile (Q3), and the whiskers represent 1.5x the IQR from Q1 and Q3. Outliers are shown as individual points. These data are from one experiment.

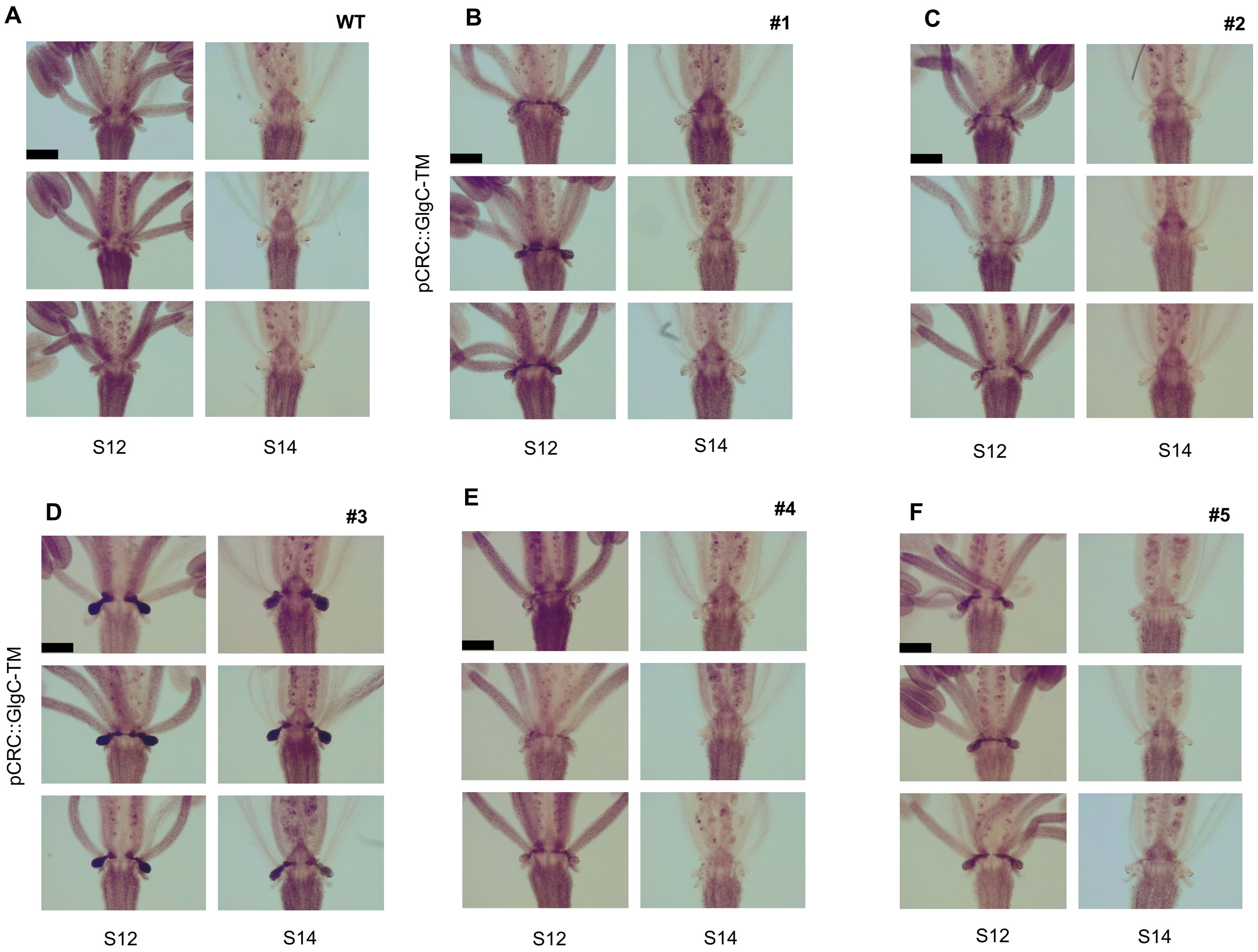

Supplementary Figure S8. All pictures from pCRC::GlgC-TM lines. Flower starch stain of (A) wild-type (WT) and (B-F) five independent pCRC::GlgC-TM lines. Floral starch was measured using a combined clear-stain protocol (iodine-based) as described in methods. Flowers were harvested at zeitgeber time 6 = ZT6 (6 hours post dawn), at indicated developmental stages (S12 = stage 12, S14 = stage 14). Each vertical set of flowers from different stages (S12, S14) were taken from the same plant to minimize plant-to-plant variation and ensure the correct stage was collected. Scale bar = 250 μm , which applies to all six images in each genotype group. These data are from one experiment.

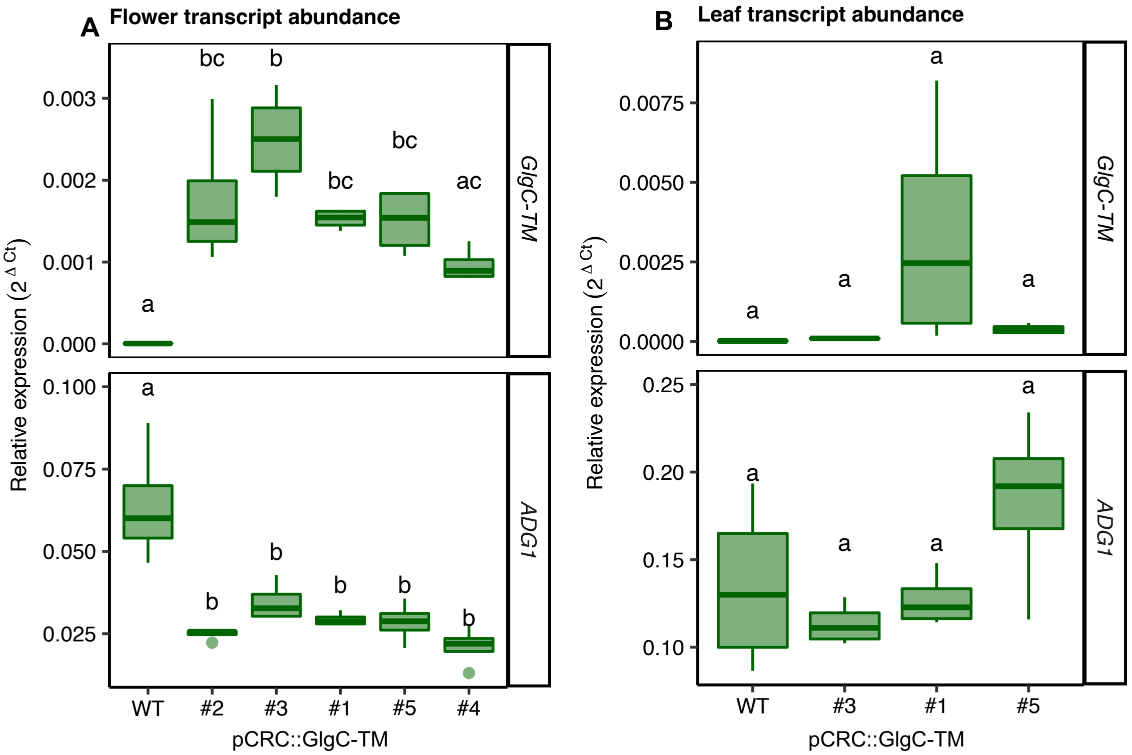

Supplementary Figure S9. pCRC::GlgC-TM lines have similar expression of *GlgC-TM* and *ADG1* in leaves and flowers. Analysis of (A) flower and (B) leaf transcript abundance for pCRC::GlgC-TM lines compared to WT. Gene expression was measured with RT-qPCR, as described in methods. Transcript abundance was measured as the number of cycles to reach a threshold fluorescence (Ct), was normalized to housekeeping gene *UBQ10*, and has been linearized for this figure ($2^{\Delta Ct}$). For A, each biological replicate represents pooled RNA of seven flowers taken from three different plants (two flowers from each of two plants, three flowers from the third). For B, each biological replicate represents the expression of one leaf taken from one plant. Boxes that share a letter are not significantly different from one another (N=4 biological replicates; One-way ANOVA with Tukey post-hoc test, $p<0.05$). The center line within each box is the median (Q2), while the box represents the interquartile range (IQR), extending from the first quartile (Q1) to the third quartile (Q3), and the whiskers represent 1.5x the IQR from Q1 and Q3. Outliers are shown as individual points. These data are from one experiment.

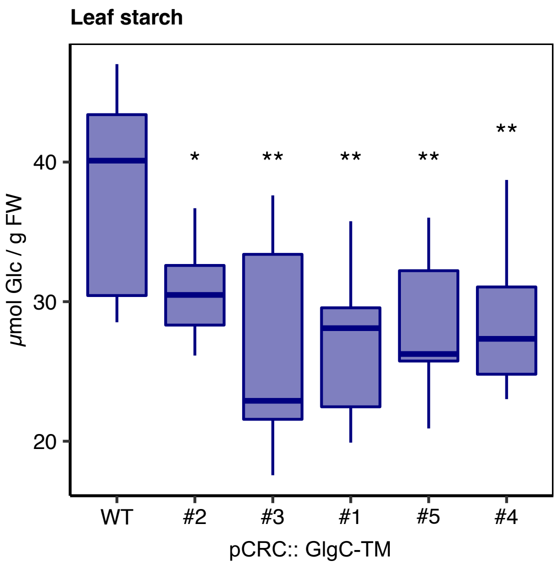

Supplementary Figure S10. Leaf starch accumulation in pCRC::GlgC-TM lines. Leaves were collected from 22-day old plants between ZT9 and ZT10, and starch was measured enzymatically (N=7; 2-sample t-test, *=p<0.1, **=p<0.05). The center line within each box is the median (Q2), while the box represents the interquartile range (IQR), extending from the first quartile (Q1) to the third quartile (Q3), and the whiskers represent 1.5x the IQR from Q1 and Q3. Outliers are shown as individual points. These data are from one experiment.

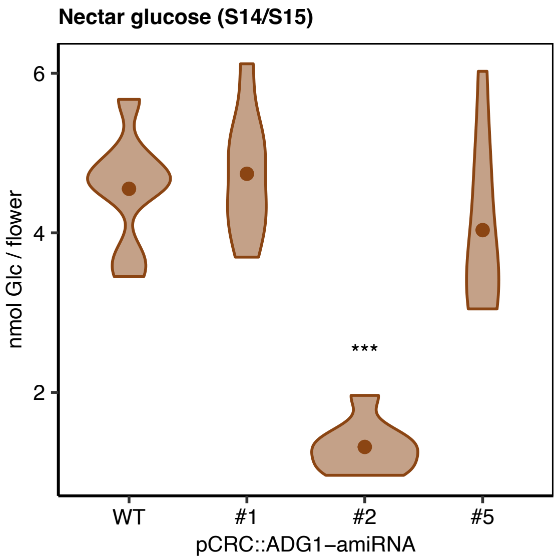

Supplementary Figure S11. Nectar sugar in pCRC::ADG1-amiRNA lines used in metabolomics experiment. Total nectar glucose (nanomoles of glucose per flower) accumulation in stage 14/15 (S14/S15) flowers collected between the hours of zeitgeber time 3 (ZT3) and ZT5. Each replicate represents a pooled sample of nectar collected from 7 flowers from 7 different plants (1 flower/plant). Violins represent distribution of the data, while points represent the average within each group (N=8; 2-sample t-test, ***=p<0.0005). These data are from one experiment.

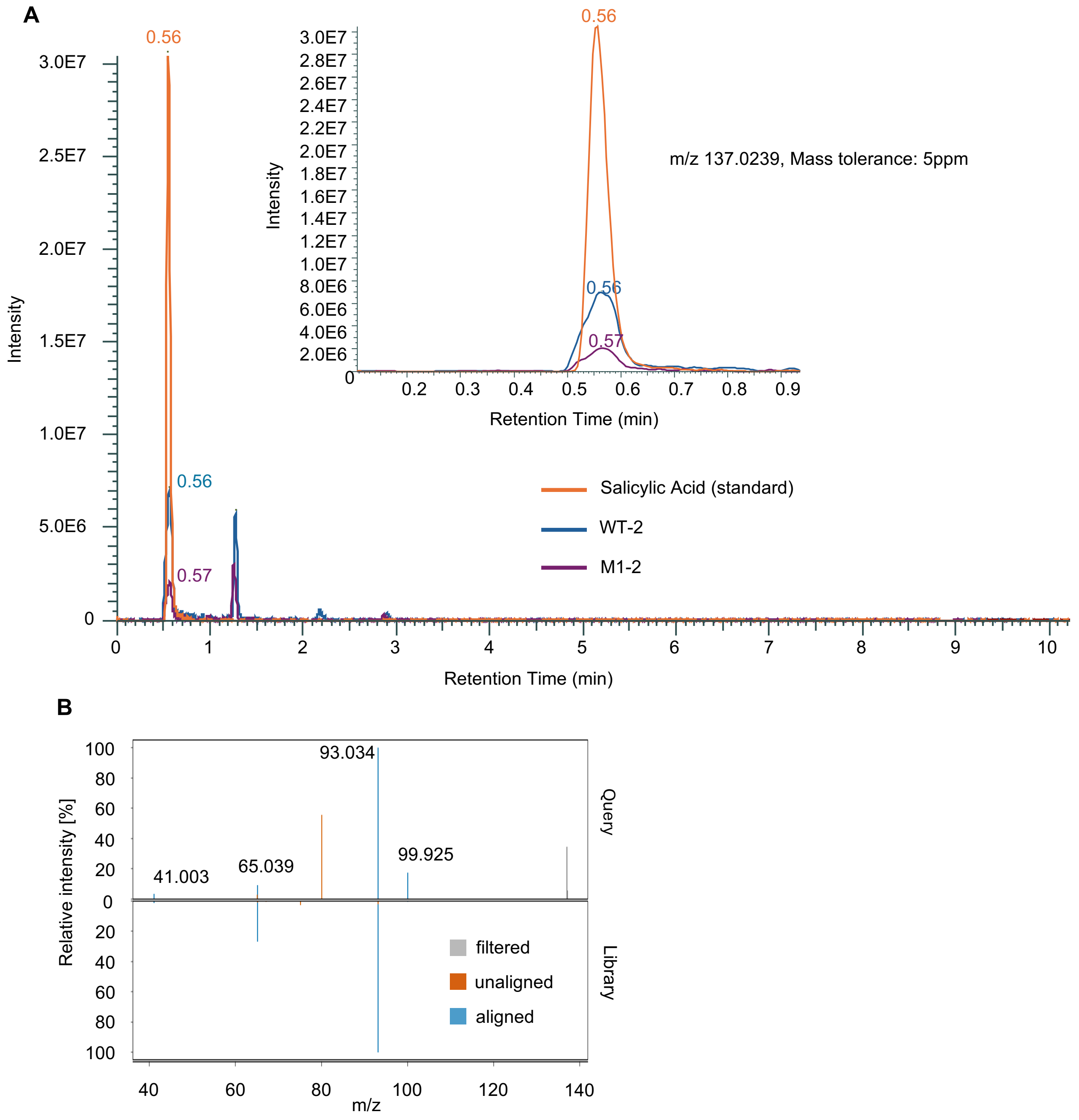

Supplementary Figure S12. Targeted validation of salicylic acid compound identification. (A-B) Targeted LC-MS/MS using standards for Salicylic acid, as described in methods. (A) Extracted ion chromatograms of an analytical salicylic acid (SA) standard compared to one wild-type (WT-2) and one pCRC::ADG1-amiRNA mutant (M1-2) sample, demonstrating similar retention times. (m/z = mass to charge ratio; ppm = parts per million). (B) MS2 mirror plot comparing the MS2 spectra of a WT sample (Query) versus a reference library (Library). Query = WT-2 sample, Library = Salicylic acid, MoNA ID = CCMSLIB00012866788 (MoNA = Mass Bank of North America). Metabolites were measured using Targeted LC/MS-MS as described in methods. Plants were grown under long-day conditions (16 h light/8 h dark) and nectar was harvested from stage 14/15 flowers at zeitgeber time 3. These data are from one experiment.

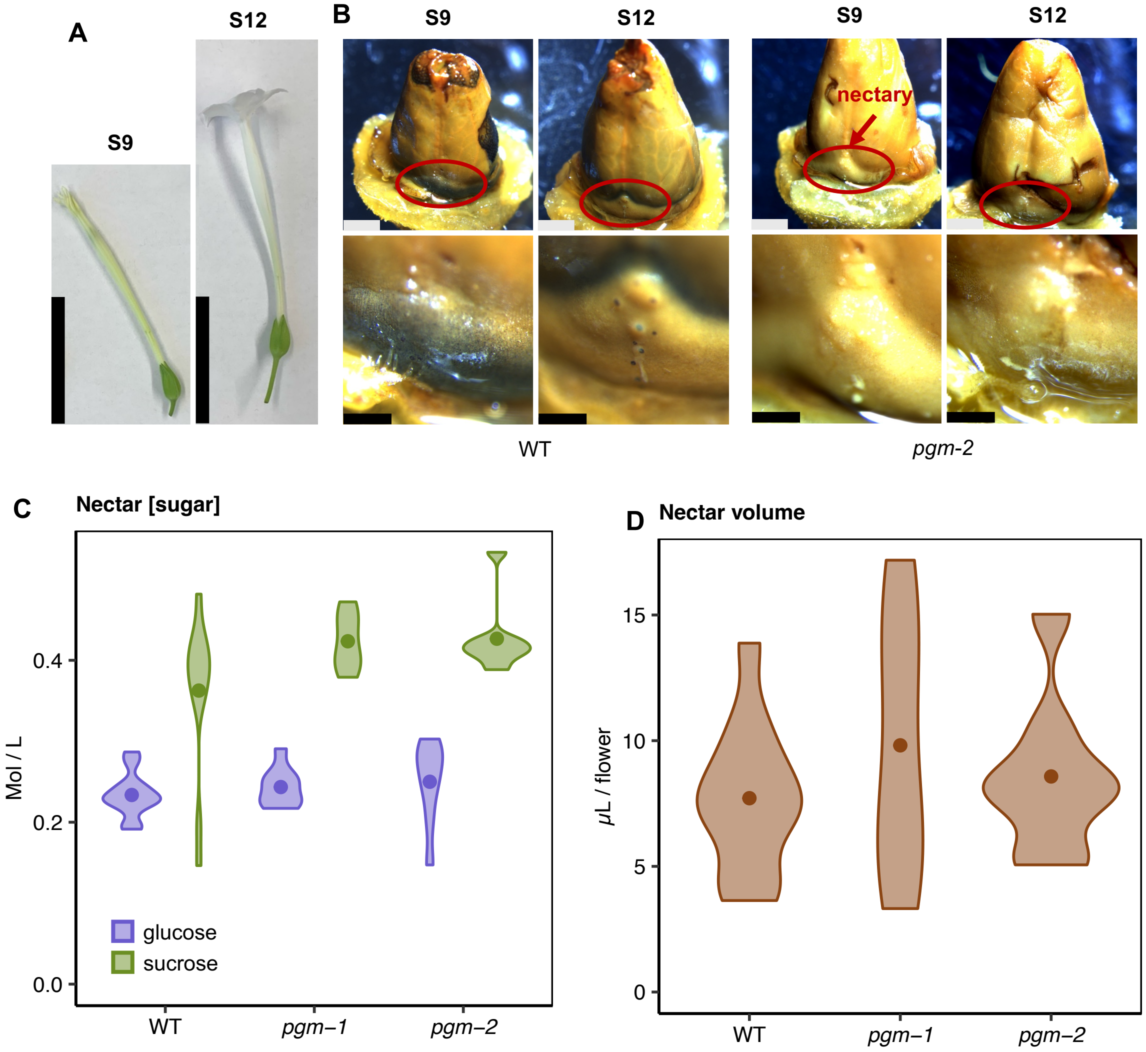

Supplementary Figure S13. Nectar volume and sugar concentration in wild-type (WT) and starchless *pgm* mutants of *Nicotiana sylvestris*. Plants were grown in greenhouse conditions, and nectar was harvested at mid-day. (A) Pictures of pre-secretory (S9) and secretory (S12) *N. sylvestris* flowers corresponding to closed and open flowers, respectively; Scale bar (black) = 5 mm. (B) Iodine staining of *N. sylvestris* flowers showing accumulation of starch in S9 nectaries followed by starch degradation during secretion (S12). Scale bar (black) = 250 μm, Scale bar (grey) = 1 mm. (C) Nectar concentration (moles per liter) of glucose (blue) and sucrose (green), measured enzymatically. (D) Nectar volume (microliter per flower), measured with microcapillary tubes. For both C and D, points represent the mean, and violins represent the distribution of the data (1 flower/replicate; N=12 for WT, N=8 for both mutants; 2-sample t-test * = $p<0.05$).

Supplementary Table S1. Primer sequences used in gene expression studies.

Name	Gene	AGI#	Sequence
AtSWEET9_F	*SWEET9*	At2g39060	CGGTGGACTTGGTCTCTTAAT
AtSWEET9_R	*SWEET9*	At2g39060	GCAAAGACTGCGAGACTGTA
AtCWINV4_F	*CWINV4*	At2g36190	GGTACATGGTCCGGTTCAATAA
AtCWINV4_R	*CWINV4*	At2g36190	GTCCTCTGGGATTGCATAGTTT
AtSPS2F_F	*SPS2F*	At5g11110	GCTCAATGCCAGGAGTCTATC
AtSPS2F_R	*SPS2F*	At5g11110	CTCTCTCCATTCTCCTGCTCTA
UBQ10_F	*UBQ10*	At4g05320	GATCCAGGACAAGGAAGGTATTC
UBQ10_R	*UBQ10*	At4g05320	GATGTTGTAGTCGGCCAAAGTA
AMY3_F	*AMY3*	At1g69830	GGGAAGACTTCTATGTCCCTTTC
AMY3_R	*AMY3*	At1g69830	TCCACTAGCAGACACTTCTTTATC
APS1_F	*ADG1*	At5g48300	TACACACAGCCGCGTTATT
APS1_R	*ADG1*	At5g48300	GTCCAACCACAGAGTGATGAA
APS1_1_Stem-loop_F	*ADG1-amiRNA*	-	CCGGCGTTAACCGTTACAAT
APS1_1_RT-loop	*ADG1-amiRNA*	-	GTCGTATCCAGTGCAGGGTCCGAGGTATTCGCACTGGATACGACGAGCGG
Sno85_Stem-loop_F	*Sno85-miRNA*		CCGGCGGTGCATTCAAAAGCC
Sno85_RT-loop	*Sno85-miRNA*		GTCGTATCCAGTGCAGGGTCCGAGGTATTCGCACTGGATACGACACATGT
AtBAM1_F	*BAM1*	At3g23920	CCGATGCTGGTCACTACAATAA
AtBAM1_R	*BAM1*	At3g23920	GGGAGTACCATGAGAGGAAGA
AtBAM9_F	*BAM9*	At5g18670	CCATGCTTTCACAGTCTTTGTC
AtBAM9_R	*BAM9*	At5g18670	CTGAACCTATGCTAGCTGTCTC
GlgC-TM_qPCR_F	*GlgC-TM*	-	GGCGGTTGATGAGAACGATAA
GlgC-TM_qPCR_R	*GlgC-TM*	-	CGTCAAAGACGTAGATACCCATAC

Supplementary Table S2. Primer sequences used in cloning.

Name	Sequence	Type
pCRC_attB4_F	GGGGACAACTTTGTATAGAAAAGTTGAAAA TCCCTTTGTCTATTCGC	cloning primer
pCRC_attB1r_R	GGGGACTGCTTTTTTGTACAAACTTGTATATGCAGTCCATAATTGCATT	cloning primer
pCRC_F1	TTGTCTATTCGCAGTTGTAAAG	sequencing primer
pCRC_R1	TACTTTGTGAGTTAAGTCCCAA	sequencing primer
pCRC_F2	ATGGAGGCGTAAGATCAATAAA	sequencing primer
pCRC_R2	GAAGTATCACAGTTCACATCAC	sequencing primer
pCRC_F3	CACCGAAACCATGATATGTAAA	sequencing primer
pCRC_R3	CCATTATTCATGCATCCACTAC	sequencing primer
pCRC_F4	TATTTCTAGTCCACGATTACCC	sequencing primer
pCRC_R4	TGGACAATGAGAAATTATCCTC	sequencing primer
pCRC_F5	TGAGAGCAAACTTCAATGTATG	sequencing primer
pCRC_R5	AAGATAGTGAAGAAAGGAGGAG	sequencing primer
pCRC_F6	AACTCAGAATCCAGCAGTATAG	sequencing primer
pCRC_R6	TCGTTTAACTGTAGAGAAAC	sequencing primer
pCRC_F7	TTGTCTTTTTGCATCGCATATG	sequencing primer
pCRC_R7	GAGAGTGAAAACAATCAATCCA	sequencing primer
pCRC_F8	AAAGTCATAAACCCCTAGATCC	sequencing primer
pCRC_R8	GTCTTTAGCGAATGGATTGAAA	sequencing primer
amiRNA_F	GACGCATATTACACATGTTCAT	sequencing primer
amiRNA_R	CTTAGTGGATCAAGCATGTTTT	sequencing primer
ADG1_attB1_F	GGGGACAAGTTTGTACAAAAAAGCAGGCTACCATGGCGTCTGTATCTGCAATT	cloning primer
ADG1_GlgC-TM_attB2_R	GGGGACCACTTTGTACAAGAAAGCTGGGTTTAATTTTATAGTTTTAACATCTTTAAAAC	cloning primer
GlgC-TM_F2	GAATTCGTTGAAAAACCTGCTA	sequencing primer
GlgC-TM_R2	CGGATTGTACGCAAGAGAG	sequencing primer
GlgC-TM_F3	GTGAATTCATTCTGCAACATTG	sequencing primer
GlgC-TM_R3	AACAAAGGAGCATTATGTGTAC	sequencing primer
